# Evaluation of Three Prokaryote Primers for Identification of Prokaryote Community Structure and Their Abode Preference in Three Distinct Wetland Ecosystems

**DOI:** 10.3389/fmicb.2021.643945

**Published:** 2021-07-14

**Authors:** Kavita Kumari, Malay Naskar, Md. Aftabuddin, Soma Das Sarkar, Bandana Das Ghosh, Uttam Kumar Sarkar, Subir Kumar Nag, Chayna Jana, Basanta Kumar Das

**Affiliations:** ^1^Aquatic Environmental Biotechnology and Nanotechnology Division, ICAR-Central Inland Fisheries Research Institute, Barrackpore, India; ^2^Fisheries Resource Assessment and Informatics Division, ICAR-Central Inland Fisheries Research Institute, Barrackpore, India; ^3^ICAR-Central Inland Fisheries Research Institute, Barrackpore, India; ^4^Reservoir and Wetland Fisheries Division, ICAR-Central Inland Fisheries Research Institute, Barrackpore, India

**Keywords:** indicator taxa, floodplain wetland, brackishwater wetland, high-throughput sequencing, sediment microbes, sewage-fed ecosystem

## Abstract

The ultimate role of prokaryote (bacteria and archaea), the decomposer of the wetland ecosystem, depends on its community structure and its interaction with the environment. The present study has used three universal prokaryote primers to compare prokaryote community structure and diversity of three distinctly different wetlands. The study results revealed that α-diversity indices and phylogenetic differential abundance patterns did not differ significantly among primers, but they did differ significantly across wetlands. Microbial community composition revealed a distinct pattern for each primer in each wetland. Overall comparison of prokaryote communities in sediments of three wetlands revealed the highest prokaryote richness and diversity in Bhomra (freshwater wetland) followed by Malencho (brackish-water wetland) and East Kolkata wetland (EKW) (sewage-fed wetland). Indicator genus analysis identified 21, 4, and 29 unique indicator genera, having preferential abode for Bhomra, EKW, and Malencho, respectively. Prediction of potential roles of these microbes revealed a preference for sulfate-reducing microbes in Malencho and methanogens in Bhomra. The distinct phylogenetic differential abundance pattern, microbial abode preference, and their potential functional role predict ecosystem variables shaping microbial diversity. The variation in community composition of prokaryotes in response to ecosystem variables can serve as the most sensitive bioindicator of wetland ecosystem assessment and management.

## Introduction

Wetlands are among the most productive ecosystems, hosting an array of ecosystem goods and services like sustaining biodiversity, ensuring livelihood, and nutritional security of millions of people (Sarkar and Borah, 2018; Sarkar et al., 2020). These wetlands play a vital role in regulating the Earth’s climate (Megonigal and Neubauer, 2019) and biogeochemical cycle (Cheung et al., 2018; Iliev et al., 2019), including organic matter decomposition, nutrient cycling, and greenhouse gas emissions (Sánchez, 2017; Zhang et al., 2019; Chandra and Kumar, 2020). The composition and functioning of microbiota—a decomposer of the ecosystem—depend highly on microbiota–environment interaction (Merkley et al., 2004; Cao et al., 2017). Their diversity and physiological dynamics are also concurrently affected by various natural and anthropogenic factors, *viz*., water table fluctuations, eutrophication, pollution, management intervention, and global warming (Jansson and Hofmockel, 2019; Zhou et al., 2020). The prokaryote (bacteria and archaea) sensitively responds to perturbations in the ecosystem; thereby, a change in its community diversity and structure can serve as the most sensitive and early bioindicator of ecosystem health assessment (Astudillo-García et al., 2019).

Extensive surveys based on next-generation high-throughput microbial 16S rRNA amplicon sequencing of various wetland sediments have revealed the potential effects of natural or anthropogenic processes on prokaryote structure at higher resolution (Adrados et al., 2014; Ansola et al., 2014; Horton et al., 2019; Li et al., 2019). More specifically, the bacterial communities are studied—on a large-scale and varied environments—more than archaeal communities, though archaea uniquely involved in methanogenic and other important pathways of the biogeochemical cycle (Offre et al., 2013; Fischer et al., 2016). Nowadays, the intense focus has shifted toward the underlying processes in sediment microbial assembly, identification of indicator taxa, and their functional properties from an initial focus typically on the description of phylum-level composition, diversity, and richness (Cao et al., 2017; Urakawa and Bernhard, 2017; De Mandal et al., 2020). Bacteria and archaea are evolutionarily and functionally diverse. And adequate wetland management requires genus-level assignment of 16S rRNA sequence data (Martens-Habbena et al., 2009; Hernández et al., 2015; Urakawa and Bernhard, 2017). Further, PCR conditions, sequencing methods, and specific short variable regions of 16S rRNA being sequenced might affect the richness and diversity of microbes of a unique ecosystem. Consequently, different sites or ecosystems must be evaluated under the same experimental conditions (Tremblay et al., 2015; Fouhy et al., 2016). Recently, Wei et al. (2019) compared China’s freshwater wetland and salt marsh to identify indicator bacterial taxa at the genus level. To the best of our knowledge, no comparative assessment of microbial diversity covering both archaea and bacteria in different wetland ecosystems is available; nor were their abode preferences identified under the same experimental conditions (Zhou et al., 2017; Wei et al., 2019; Zhao et al., 2020). Further, no evidence is available on the evaluation of widely used universal prokaryote primers for their use in distinct wetland ecosystems.

India has a wealth of wetland ecosystems covering an area of 1.33 million ha that support diverse and unique biodiversity. These wetlands are facing challenges emerging from both natural and anthropogenic disturbances for years (Raina et al., 2019; De Mandal et al., 2020; Sarkar et al., 2020). Research studies addressed the diversity and functional aspects of microbes in wetland ecosystems to a limited extent in the Indian context (Rathour et al., 2017; Raina et al., 2019; De Mandal et al., 2020). Therefore, the present study has worthily carried out a comparative analysis of microbial taxa (including bacteria and archaea) among three distinctly different wetland ecosystems (fresh, brackish, and sewage-fed)—under the same analytical condition. Three widely used universal prokaryote primers were evaluated for microbial identification of each wetland sediment. This research will provide the first evidence on the comparative evaluation of these primers, consequently bringing out more insights into the prokaryote profile involved in the biogeochemical cycle in distinct wetland ecosystems. The specific aim of the study is to evaluate three widely used universal prokaryote primers for (i) investigating microbial diversity, and community composition of three wetland sediments, and (ii) predicting microbial abode preference along with wetland-specific microbial indicators.

## Materials and Methods

### Description of Study Site

**FIGURE 1:**
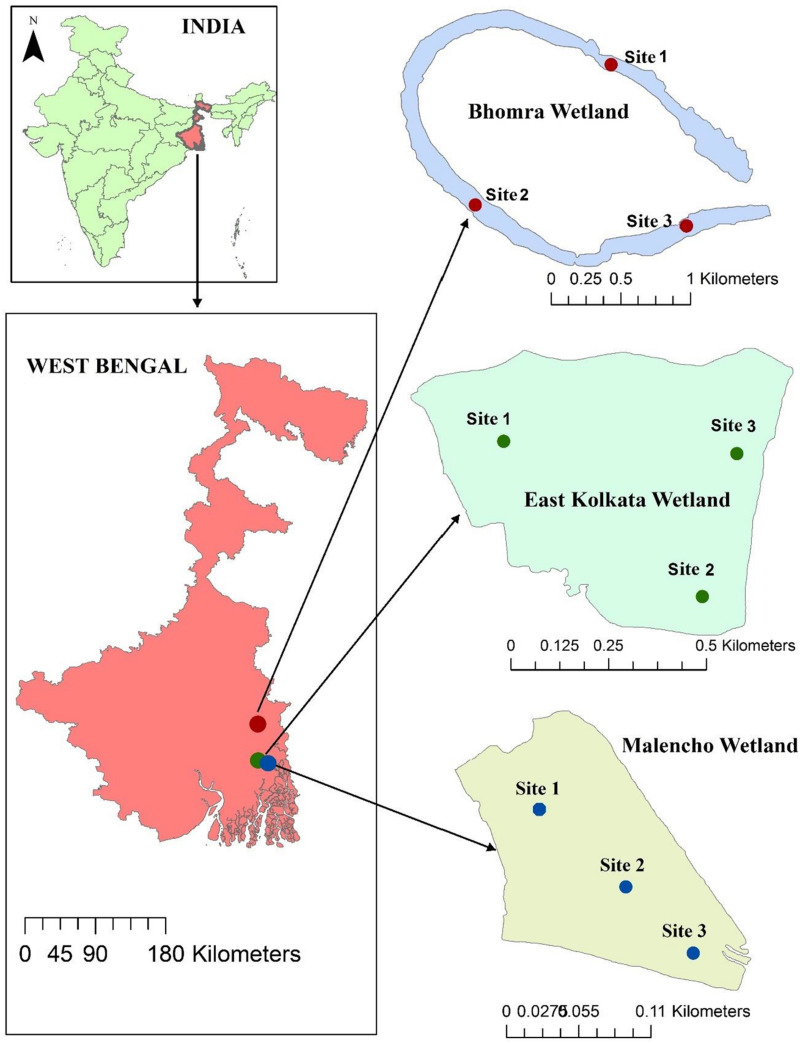
Geographical location of each sampling site in three different wetland habitats.

The study area belongs to West Bengal, India. Three distinct wetlands—a sewage-fed wetland (East Kolkata wetland; hereafter abbreviated as EKW), a freshwater wetland (Bhomra), and a brackish-water wetland (Malencho)—were selected from the study area (Figure 1). EKW, located in North 24 Pargana district, is one of the most unique sewage-fed natural wetland systems in the country, with an area of 12,500 ha. It has high conservation significance, getting designated as a Ramsar site (Ramsar Site No. 1208), and is recognized as the world’s largest sewage-fed aquaculture system. It provides a low-cost, efficient, and environmental friendly solid-waste and sewage treatment system for the city of Kolkata (Ghosh, 1998; Nag et al., 2019). EKW also supply water to nearby urban agricultural activities (Roy-Basu et al., 2020). The present study was conducted in one unit of EKW (22°59′14.59″N; 88°37′40.33″E) with a water surface area of 55 ha. Bhomra is a perennial and oxbow-shaped floodplain wetland—locally known as “Bhomra beel.” It is situated in the lower Gangetic plain (22°59′14.59″N; 88°37′40.33″E), Nadia district, with a water surface area of 45.7 ha (Figure 1). This wetland is classified as seasonally open and receives monsoon flood pulses from the river Ichhamati, a trans-boundary river between India and Bangladesh. The Malencho (Figure 1; 22°27′23.21″N; 88°18′43.91″E) is a brackish-water wetland—locally known as “Bheri” (man-made impoundments in coastal areas)—characterized by its alternate crop (rice and fish) cultivation practices. With a water surface area of 90 ha, the wetland is situated in the South 24 Pargana district. This ecosystem receives saline influx during high tide from the adjoining areas through sluices and is suitably used for polyculture of shrimp and fish species without incurring additional operational costs (Ghoshal et al., 2019).

### Collection of Water and Sediment Samples

Sediment and water samples were collected from the selected wetlands during December–February 2017. Sediment samples were taken in triplicate from three sectors of the wetlands at the 15 cm of depth using Peterson grab to represent all types of microhabitats (Andrade et al., 2012). All the nine samples were pooled and thoroughly mixed before they were sieved through 2.0-mm mesh. The collected sediment samples were transferred immediately to a sterile plastic container and transported to the laboratory on ice within 6 h. Samples were subsequently divided into two parts. One part was air-dried under shade for analysis of physico-chemical properties, and the counterpart was stored at −20°C for molecular analysis of microbes. Water samples were collected from the subsurface layer using a standard water sampler (Das Sarkar et al., 2019).

### Physico-Chemical Characterization of Water

Physico-chemical parameters of water—temperature, pH, specific conductivity, and salinity—were determined with a multi-parameter Testr^TM^ 35 series (Eutech Instruments, Oakton, Singapore). Transparency of the water was measured using a 20-cm-diameter standard black and white Secchi disk (Strickland and Parsons, 1972). Further, the parameters—dissolved oxygen (DO; APHA, 2017), free carbon dioxide (free CO_2_), total alkalinity, total hardness, available nitrate-N (NO_3_^–^), and available phosphate-P (PO_4_^–^)—were determined following standard estimation processes (APHA, 2005). Chlorophyll *a* was estimated using the acetone extraction method and thereafter calculated from measured optical density (Jeffrey and Humphrey, 1975). The trophic state index (TSI) was estimated following Das Sarkar et al. (2020). Total organic carbon (TOC) and total inorganic carbon (TIC) in water samples (Urbansky, 2001) were determined in a TC Analyzer (OI Analytical, Model-Aurora 1030; Xylem Inc., Rye Brook, NY, United States).

### Sediment Quality Parameter

Standard procedures were followed for the determination of soil pH (Thomas, 1996), specific conductivity (Rhoades, 1996), and total phosphorus (Olsen and Dean, 1965). Organic carbon (C) content of sediment was determined by dry combustion method at 900°C (Nelson and Sommers, 1996), and estimation of C was done using TC Analyzer (OI Analytical, Model-Aurora 1030; Xylem Inc., United States).

### Isolation and Purification of DNA

DNA was isolated from sediment samples using Macherey-Nagel (Düren, Germany) NucleoSpin^®^ Soil Kit according to the manufacturer’s instructions. Approximately 0.25 g of homogenized sediment was used for the isolation of genomic DNA. The quality of the extracted DNA was evaluated on 1% agarose gel, and the concentration of DNA was measured with a NanoDrop spectrophotometer (ND-1000; Isogen Life Science, De Meern, Netherlands).

### Illumina-Based Sequencing of 16S rRNA Gene Amplicon

High-throughput paired-end sequencing of the prokaryote 16S rRNA gene was performed on the Illumina (San Diego, CA, United States) MiSeq platform. Briefly, the V3–V4 region of prokaryote 16S rRNA gene was amplified by the primer pair 341b4_F and 806_R (Lu et al., 2015), primer pair Pro341F and Pro805R (Takahashi et al., 2014), and modified primer pair N341b4_F and 806_R (present study; Supplementary Table 1). The modified primer pair in the present study was used to cover Verrucomicrobia and Opitutae groups, as it previously showed biases toward these groups (Takahashi et al., 2014; Lu et al., 2015). Further, three universal prokaryote primers covering both V3 and V4 regions were used to maximize taxonomic coverage and target specificity (Wilcox et al., 2013; Thijs et al., 2017; Schriefer et al., 2018).

Briefly, PCR was performed in 25 μl of mixture containing 5.0 μl of forward primer (1 μM), 5.0 μl of reverse primer (1 μM), 2.5 μl of DNA template (10 ng/μl), and 12.5 μl of 2 × KAPA HiFi HotStart ReadyMix (KAPA Biosystems, Woburn, MA, United States). The thermal program setting was as follows: 95°C for 3 min; 25 cycles of 95°C for 30 s, 55°C for 30 s, and 72°C for 30 s; and a final extension at 72°C for 5 min. PCR products were purified using an AMPure XP bead (Beckman Coulter, Brea, CA, United States) followed by second step PCR to attach dual indices and Illumina sequencing adapters using the Nextera XT Index Kit. Briefly, PCR was performed in 50 μl of mixture containing 5 μl of DNA, 5 μl of Nextera XT Index Primer 1 (N7xx), 5 μl of Nextera XT Index Primer 2 (S5xx), 25 μl of 2 × KAPA HiFi HotStart ReadyMix (KAPA Biosystems), and 10 μl of PCR-grade water. The thermal program was as follows: 95°C for 3 min; 8 cycles of 95°C for 30 s, 55°C for 30 s, and 72°C for 30 s; and a final extension at 72°C for 5 min followed by PCR cleanup using AMPure XP beads and Illumina MiSeq sequencing (2 × 300 bp).

### Bioinformatics Processing

**FIGURE 2:**
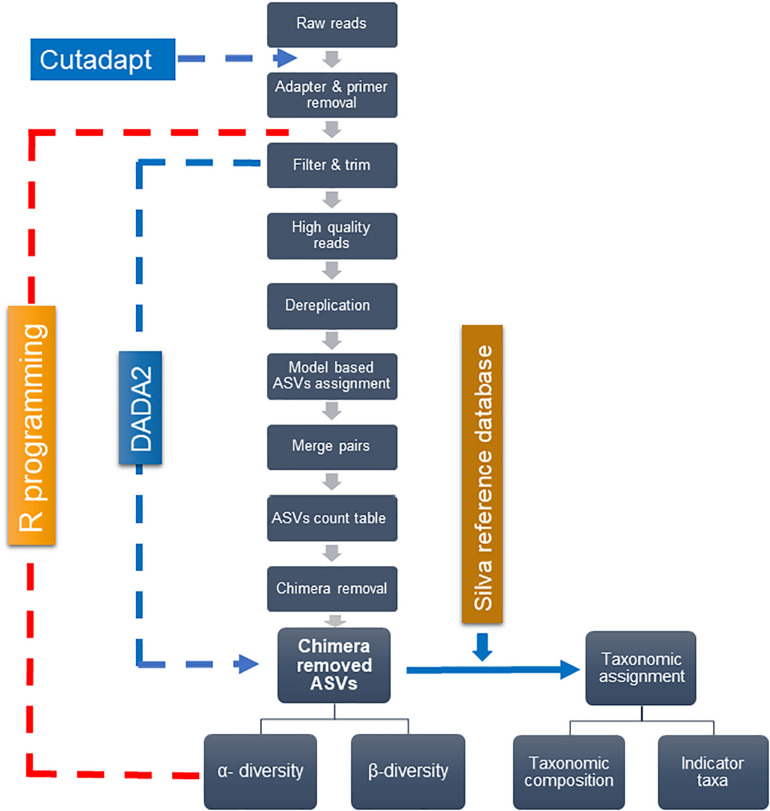
Analytical pipeline for main bioinformatics steps.

Figure 2 depicts the analytical framework of bioinformatics processing. First, the adapter and primers were removed from de-multiplexed raw pair-end reads–forward and reverse, using “*cutadapt*” (Martin, 2011). The core bioinformatics processing was then carried out, using DADA2 (Callahan et al., 2016) under R software (R Core Team, 2019). High-quality reads were retained through filtering and trimming, and low-quality reads to a consistent length. The filtering and trimming were done by setting truncation length of 284 and 200 bases for forward and reverse reads, respectively, and the maximum of one expected error. Dereplication further removed redundant sequences. A typical workflow further clusters sequence reads into operational taxonomic units (OTUs) with a fixed dissimilarity threshold. Instead, we used DADA2 method, which does not impose any arbitrary threshold, and thereby infer amplicon sequence variants (ASVs) more precisely (Callahan et al., 2016). This method relies on the model-based substitution of errors estimated from the sequence data itself. The “*learnErrors*” function generated the errors that were further plugged-in into the “*DADA*” function, inferring ASVs separately for forward and reverse sequences. The “*mergePairs*” function was used to merge forward and reverse ASVs to obtain the target length of 426 bp—with the typical overlap of 58 bp and a minimum overlap of 30 bps. The ASV count table was generated with the “*makeSequenceTable*” function, which was further refined by removing chimera using the “*removeBimeraDenovo*” function. Finally, the “*assignTaxonomy*” function, which uses naïve Bayesian classifier method (Wang et al., 2007), assigned taxonomy to ASVs—using the SILVA reference database (Quast et al., 2013) with 97% identity.

Based on the ASV count table, the data analysis set off with α-diversity measures—the rarefaction curve, estimates of richness (observed ASVs and expected ASVs), and diversity indices (Shanon and inverse Simpson)—using “*vegan*” (Oksanen et al., 2015) package under R software. An approximate pairwise test was performed to examine significant differences in the expected number of ASVs between primers within each wetland. Further, two-way ANOVA was applied to examine differences in diversity indices across wetlands and primers. The dominance pattern of ASVs was compared by “*k-dominance*” curves generated by plotting the cumulative proportion of abundances up to the k-th rank of the ASV.

Exploratory ordination technique—namely, non-metric multidimensional scaling (NMDS)—was used to compare the prokaryote community structure among samples. Two dissimilarity measures—weighted UniFrac (Lozupone et al., 2007) and abundance-based Bray–Curtis—were used for NMDS. The phylogenetic tree required to compute UniFrac was built with the “*phangorn*” package (Schliep et al., 2017), in R software; and it was then used with the “*phyloseq*” (McMurdie and Holmes, 2013) package of R. Further, analysis of similarity (ANOSIM) examined the statistical significance of dissimilarity among samples (primers and wetlands).

### Analysis of Indicator Taxa

An indicator taxa analysis technique was used to diagnose the indicator taxa (phylum and genus levels), using two types of indices: “*correlation*” (Tichý and Chytrý, 2006) and “*IndVal*” (Dufrêne and Legendre, 1997). The correlation indices determined the taxon’s abode preference to a particular wetland, and the “*IndVal*” identified the diagnostic or bioindicator taxon for a particular wetland. The indicator taxa analysis was carried out by using the library “*indispecies*” (De Cáceres and Legendre, 2009) under the R software environment, which diagnosed two types of indicator taxa: single and second-order combination of taxa. The permutation test with 1,500 repetitions determined the statistical significance of both indices. The “*IndVal*” consists of two components: the positive predicting value of a taxon as an indicator (A) of a wetland in the present case and the positive value that determines how easy it is to detect an ASV (B) in a wetland. Dufrêne and Legendre (1997) argued that a taxon with high values of A and B is easy to detect as well as to predict, qualifying as a good bioindicator. But the taxon with either high A or high B refers to asymmetrical indicator taxon. The species level similarity search for indicator genus was conducted using National Center for Biotechnology Information (NCBI), Basic Local Alignment Search Tool (BLAST), against the 16S ribosomal database.

## Results

### Physico-Chemical Properties of Water and Sediment

The physico-chemical properties of water and sediment samples of the three wetlands varied considerably (Table 1). The wetland Malencho was found to be more saline in comparison with EKW and Bhomra. Among the two freshwater wetlands, EKW showed more conductivity due to the regular intake of sewage. The nutrients phosphate and nitrate were also the lowest in Bhomra compared with EKW, whereas the highest phosphate was observed in Malencho and nitrate in EKW. The TSI values revealed a higher trophic state in EKW and Malencho than Bhomra. Bhomra has the highest water TOC, followed by Malencho and EKW, whereas the lowest TIC was observed in Bhomra and highest in EKW. The average water pH varied from 7.94 to 9.14, with the highest and lowest values in Bhomra and EKW, respectively. With respect to sediment parameters, the average pH varied from 7.58 to 8.00, with the highest and lowest values observed in Bhomra and Malencho, respectively. Carbon content was noticeably the highest in Bhomra followed by nearly equal and low levels in Malencho and EKW. Specific conductivity was high in EKW and Malencho and low in Bhomra. The highest level of total phosphorus was detected in EKW and the lowest in Malencho.

**TABLE 1:** Physico-chemical parameters (mean ± SD) of water and sediment samples in Bhomra (freshwater), EKW (sewage-fed), and Malencho (brackish-water) wetlands.

Parameters (units)	Wetlands		
	Bhomra	EKW	Malencho
**Water**			
Salinity (mg/L)	195.33 ± 1.2	375.33 ± 1.17	526.33 ± 1.15
Air temperature (°C)	25.03 ± 0.015	23.70 ± 0.1	31.47 ± 0.1
Water temperature (°C)	23.60 ± 0.1	22.70 ± 0.1	30.53 ± 0.085
Depth (cm)	266.09 ± 32.38	51.82 ± 0.51	74.06 ± 4.0
Transparency (cm)	185.01 ± 0.2	34.00 ± 0.2	49.67 ± 0.42
pH	9.14 ± 0.02	7.94 ± 0.05	8.75 ± 0.05
Dissolved oxygen (mg/L)	9.40 ± 0.11	11.30 ± 0.25	13.20 ± 0.2
Total hardness (mg/L)	132.33 ± 1.15	110.67 ± 0.53	135.00 ± 0.76
Total alkalinity (mg/L)	210.33 ± 0.52	196.67 ± 0.9	183.33 ± 1.018
Free CO_2_ (mg/L)	2.67 ± 0.17	4.70 ± 0.057	4.40 ± 0.057
Specific conductivity (μS/cm)	383.00 ± 7.5	763.67 ± 12.52	937.00 ± 10.78
Available phosphate-P (mg/L)	0.01 ± 0.001	0.06 ± 0.005	0.09 ± 0.002
Available nitrate-N (mg/L)	0.14 ± 0.01	0.59 ± 0.02	0.17 ± 0.01
Chlorophyll *a* (mg/L)	0.0042 ± 0.002	0.133 ± 0.002	0.14 ± 0.002
TOC (ppm)	7.72 ± 0.12	4.302 ± 0.1	4.742 ± 0.1
TIC (ppm)	27.24 ± 0.24	44.211 ± 0.2	38.989 ± 0.67
Trophic state index	27.09 ± 0.33	55.33 ± 1.5	55.73 ± 0.1

**Trophic status**	**Oligotrophic**	**Eutrophic**	**Eutrophic**

**Sediment**			
pH	8.0 ± 0.1	7.85 ± 0.05	7.58 ± 0.076
Specific conductivity (μS/cm)	441 ± 4.51	864.33 ± 4.16	814.21 ± 4.50
Total phosphorus (mg/kg)	642.03 ± 7.5	733.59 ± 5.31	33.11 ± 0.50
Organic carbon (%)	6.48 ± 0.061	2.8 ± 0.08	2.49 ± 0.07

*EKW, East Kolkata wetland; TOC, total organic carbon; TIC, total inorganic carbon.*

### Read Output and Parametric Analyses of Illumina MiSeq Pyrosequencing Data

The Illumina MiSeq sequencing generated a total of 3,502,469 raw reads for the nine samples—the average number of reads per sample was 389,163, ranging between 262,163 and 461,999. Further bioinformatics processing retained a total of 1,277,354 reads, ranging between 93,436 and 209,785 (Supplementary Table 2).

**FIGURE 3:**
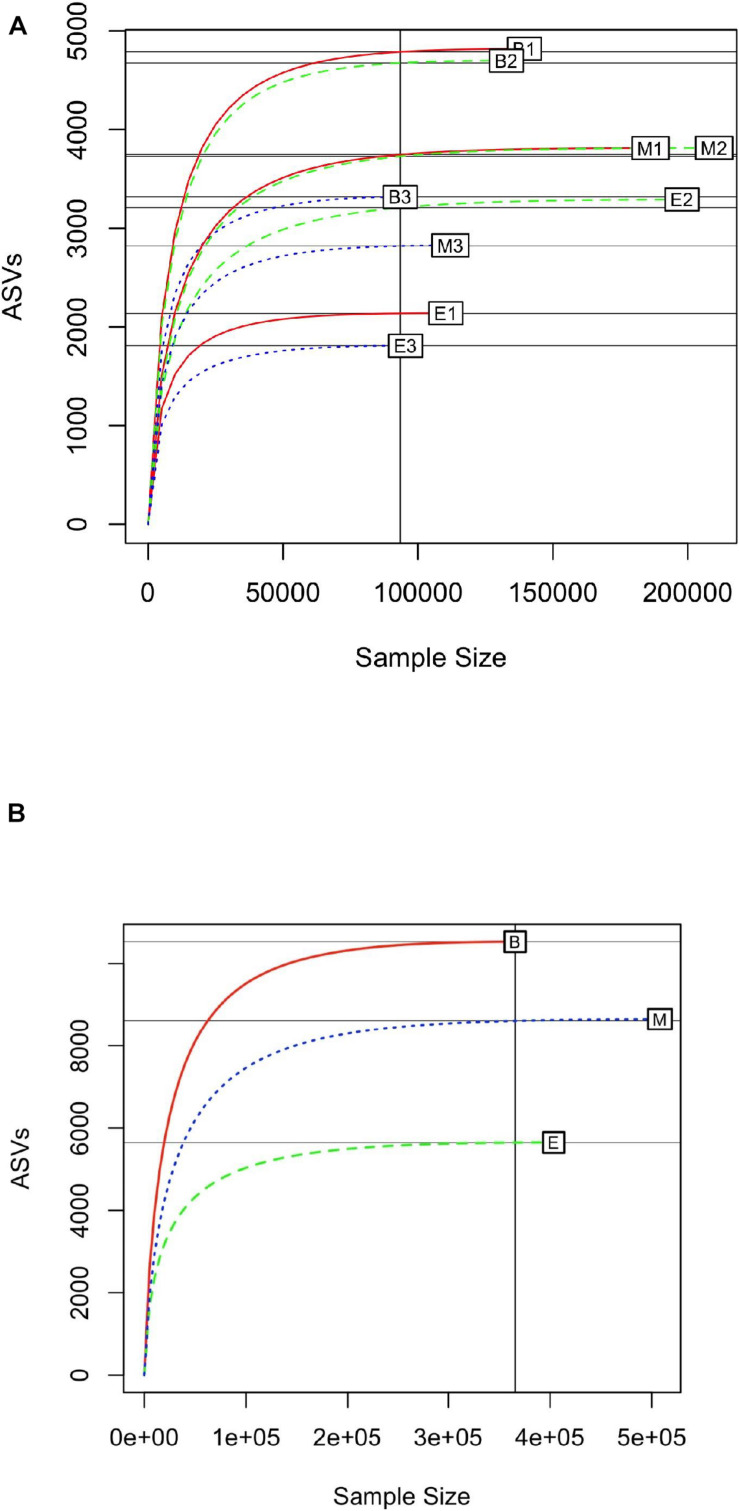
**(A)** Rarefaction curve of 16S prokaryote sequences detected by three universal prokaryote primers (341b4_F/806_R, N341b4_F/806_R, and Pro341F/Pro805R) in Bhomra (freshwater), EKW (sewage-fed), and Malencho (brackish-water) wetlands. The abbreviations and numerics used are as follows: B, Bhomra; E, EKW; M, Malencho; 1, 341b4_F/806_R; 2, N341b4_F/806_R; 3, Pro341F/Pro805R; EKW, East Kolkata wetland. **(B)** Rarefaction curve of 16S prokaryote sequences detected in three wetland sediments (Bhomra, EKW, and Malencho). The data are pooled over three primers in each wetland. The abbreviations used are as follows: B, Bhomra; E, EKW; M, Malencho.

Rarefaction curves were generated with a subsample size of 93,346, to identify the minimum number of observed ASVs in all samples. In general, the primer 341b4_F/806_R showed the steepest slope, followed by N341b4_F/806_R and Pro341F/Pro805R (Figure 3A); and among the wetlands, Bhomra showed the steepest slope, followed by Malencho and EKW (Figure 3B). In EKW, primer N341b4_F/806_R showed the steepest slope, followed by N341b4_F/806_R and Pro341F/Pro805R. The rarefaction curve attained saturation of around 70,000 sequence reads for all the samples signifying the sequencing depth adequacy. The estimated expected number of ASVs (with a random subsample size of 70,000) was 1,797 ± 4 to 4,719 ± 9 for nine samples (Supplementary Table 3).

**FIGURE 4:**
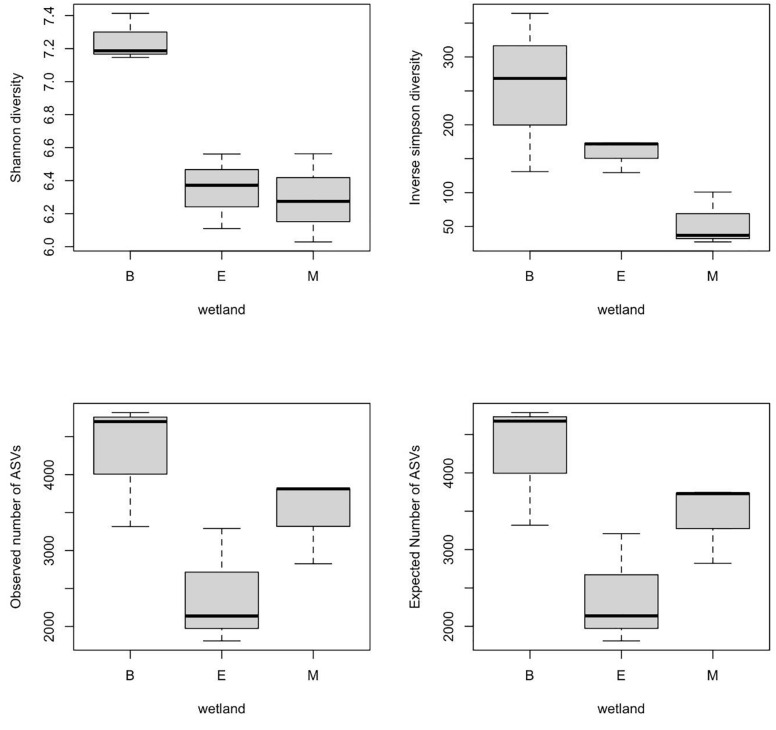
Wetland-wise comparison of different prokaryote ASV α-diversity indices (Shanon and inverse Simpson) and richness (observed ASVs and expected ASVs). The data are pooled over three primers for each wetland. The abbreviations used are as follows: B, Bhomra; E, EKW; M, Malencho; ASV, amplicon sequence variant; EKW, East Kolkata wetland.

**FIGURE 5:**
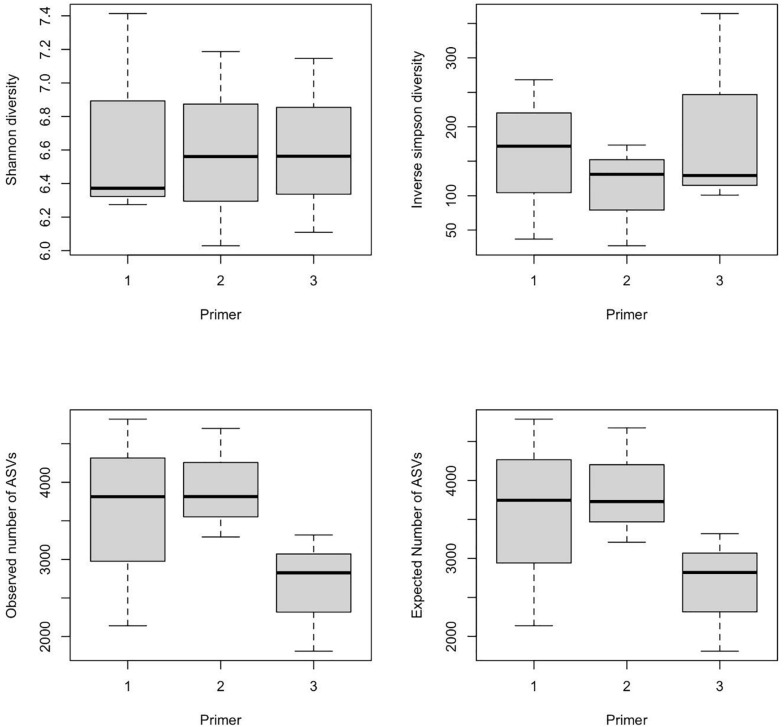
Primer-wise comparison of different prokaryote ASV α-diversity indices (Shanon and inverse Simpson) and richness (observed ASVs and expected ASVs). The data are pooled over three wetlands for each primer. The numerics and abbreviations used are as follows: 1, 341b4_F/806_R; 2, N341b4_F/806_R; 3, Pro341F/Pro805R; ASV, amplicon sequence variant.

α-Diversity indices of ASVs indicated that diversity indices did not differ significantly with primers; however, they were significantly different across wetlands (ANOVA, *p*-value < 0.05). Specifically, the Shannon index of Bhomra was significantly higher than that of EKW and Malencho, and similar results were obtained using inverse Simpson’s index (Figure 4). Concerning the ASV richness and the number of observed and expected ASVs, EKW was significantly lower than the other two wetlands (Figure 4). Diversity indices were statistically insignificant among primers (Figure 5).

**FIGURE 6:**
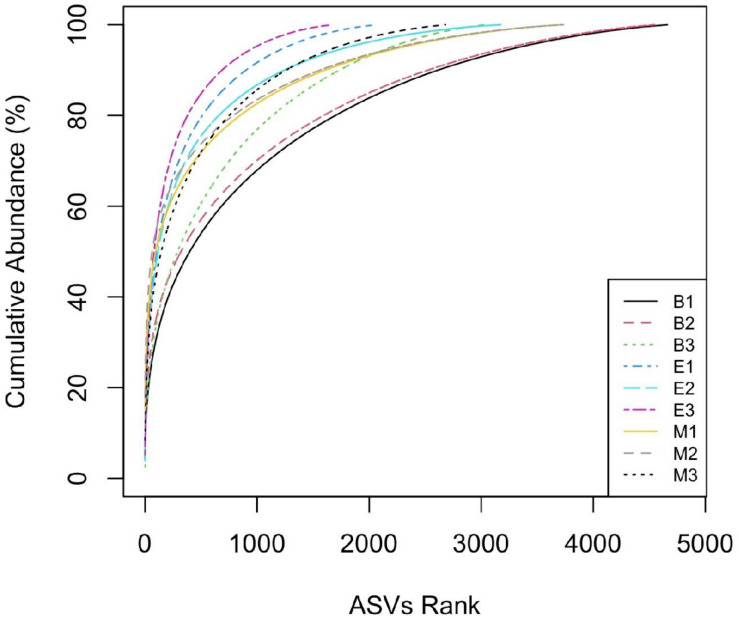
Comparative “k-dominance” curve to predict dominance pattern of ASVs for three universal prokaryote primers in each wetland. The abbreviations and numerics used are as follows: B, Bhomra; E, EKW; M, Malencho; 1, 341b4_F/806_R; 2, N341b4_F/806_R; 3, Pro341F/Pro805R; ASV, amplicon sequence variant; EKW, East Kolkata wetland.

The “*k-dominance*” curve (Figure 6) indicated fewer ASVs dominating the abundance distribution of primer Pro341F/Pro805R than those of the other two primers for all the wetlands. For example, ASVs ranked up to 1,000 accounted for 95%, 91%, and 87% of ASVs, respectively, for Pro341F/Pro805R, 341b4_F/806_R, and N341b4_F/806_R in EKW.

### Prokaryote Community Structure

**FIGURE 7:**
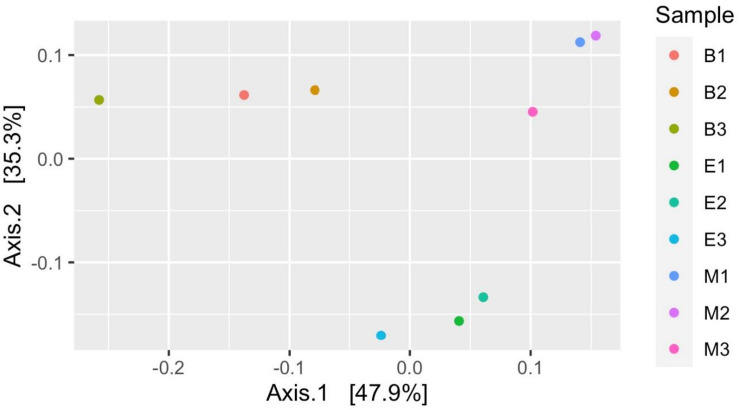
Prokaryote community structure (ASV level) among samples based on weighted UniFrac distance (phylogenetic relatedness). Each sample is represented as a dot and is colored distinctly based on wetland sediment and primer combination. The abbreviations and numerics used are as follows: B, Bhomra; E, EKW; M, Malencho; 1, 341b4_F/806_R; 2, N341b4_F/806_R; 3, Pro341F/Pro805R; ASV, amplicon sequence variant; EKW, East Kolkata wetland.

**FIGURE 8:**
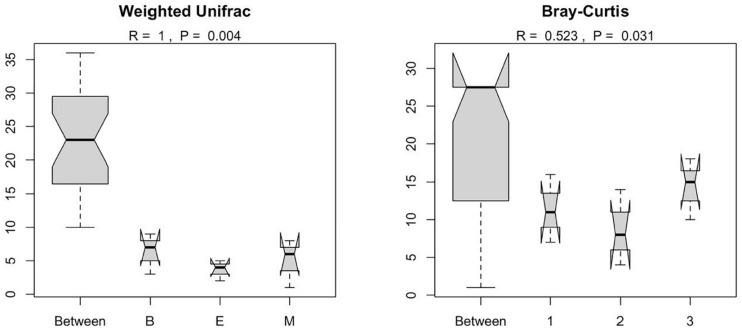
Results of ANOSIM showing the statistical significance of differential prokaryote community structure (based on weighted UniFrac and Bray–Curtis) between wetlands and primers; The abbreviations and numerics used are as follows: B, Bhomra; E, EKW; M, Malencho; 1, 341b4_F/806_R; 2, N341b4_F/806_R; 3, Pro341F/Pro805R; EKW, East Kolkata wetland.

**FIGURE 9:**
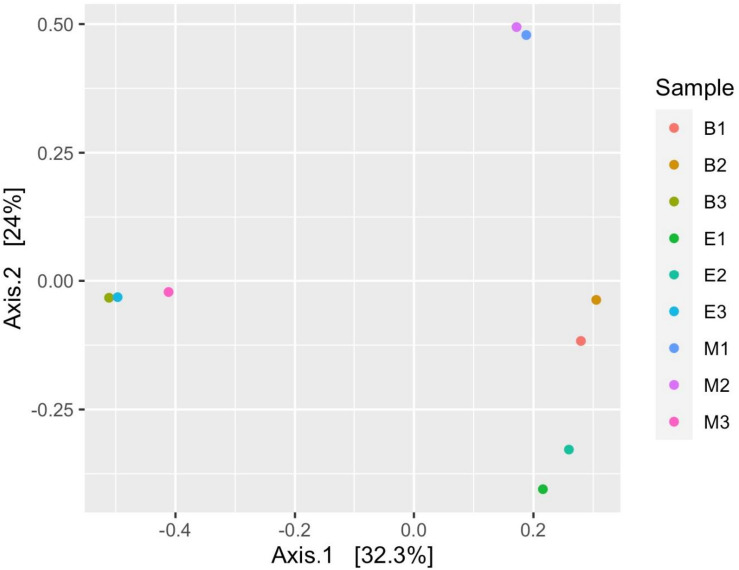
Prokaryote (ASV level) compositional dissimilarity among samples, based on the Bray–Curtis dissimilarity (non-phylogenetic relatedness). Each sample is represented as a dot and is colored distinctly based on wetland sediment and primer combination. The abbreviations and numerics used are as follows: B, Bhomra; E, EKW; M, Malencho; 1, 341b4_F/806_R; 2, N341b4_F/806_R; 3, Pro341F/Pro805R; ASV, amplicon sequence variant; EKW, East Kolkata wetland.

Figure 7 suggests dissimilar community composition among wetlands but similar community composition among primers, according to phylogenetic relatedness (weighted UniFrac). Separate ANOSIM for wetlands and primers further ensured the statistical significance of those dissimilarities (Figure 8). Based on the analysis of the Bray–Curtis dissimilarity, a differential non-phylogenetic abundance pattern existed among the primers, which was statistically significant (Figure 9). Specifically, the community composition of the primer Pro341F/Pro805R evinced a quite dissimilar composition to the two primers (Figure 9). However, wetlands were not significantly different in community composition based on pool data over primers, albeit visual inspection suggests compositional dissimilarity between Malencho and the other two wetlands (Figure 9).

### Phylum-Level Taxonomic Distribution

**FIGURE 10:**
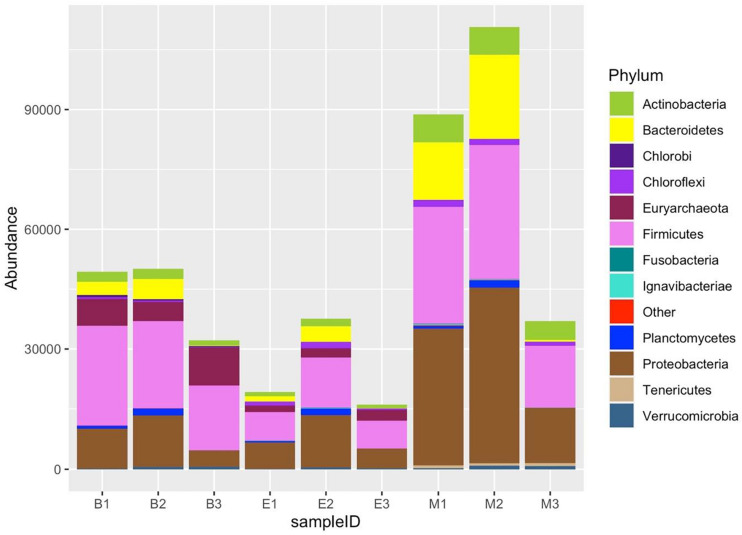
Twelve most abundant phyla, detected by three universal prokaryote primers in each wetland. The remaining taxa with lower abundance are classified into “Other.” The abbreviations and numerics used are as follows: B, Bhomra; E, EKW; M, Malencho; 1, 341b4_F/806_R; 2, N341b4_F/806_R; 3, Pro341F/Pro805R; EKW, East Kolkata wetland.

All the nine samples consisted of 47 phyla. The 12 most abundant phyla included Proteobacteria, Firmicutes, Acidobacteria, Euryarchaeota, Actinobacteria, Chloroflexi, Bacteroidetes, Aminicenantes, Planctomycetes, Crenarchaeota, Cyanobacteria, and Verrucomicrobia (Figure 10). Of the top 12 abundant phyla, Proteobacteria, Acidobacteria, Chloroflexi, Planctomycetes, Cyanobacteria, and Verrucomicrobia were more abundant in EKW. The relative abundance of Euryarchaeota, Aminicenantes, and Crenarchaeota was more in Bhomra, while the relative abundance of Firmicutes, Bacteroidetes, and Actinobacteria was more in Malencho.

Proteobacteria was the most dominant phylum in all wetlands. Firmicutes was the second most abundant phylum in Bhomra and Malencho, whereas Acidobacteria was the second most abundant phylum in EKW. Primer-specific differences were observed in many higher-abundance group phyla (Supplementary Table 4). Additionally, primer-specific difference was noticed in the case of many low abundant phyla (Supplementary Table 4)—an abundance of < 1% in all the samples.

Sharing pattern of primers showed primers—341b4_F/806_R, N341b4_F/806_R, and Pro341F/Pro805R—accounted for more than 90% of the ASVs. Wetland-specific phyla sharing was as follows: 32 in Bhomra and 30 in EKW and Malencho (Supplementary Figure 1).

### Genus and Amplicon Sequence Variant Level Analysis

**FIGURE 11:**
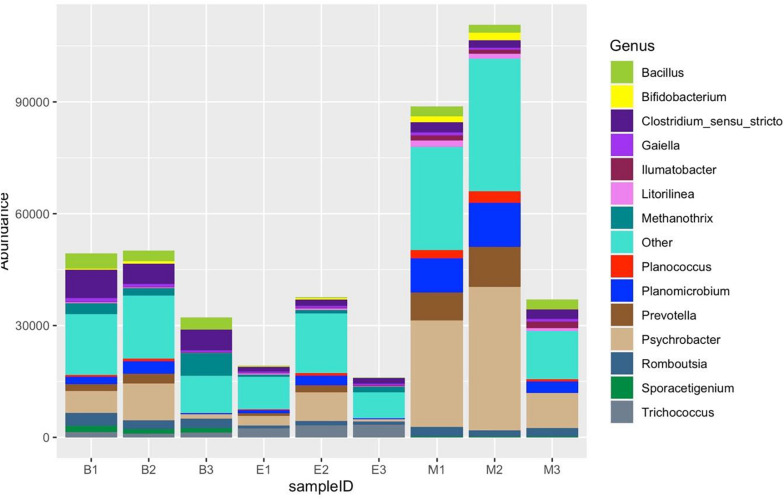
Fourteen most abundant genera, detected by three universal prokaryote primers in each wetland. The remaining taxa with lower abundance are classified into “Other.” The abbreviations and numerics used are as follows: B, Bhomra; E, EKW; M, Malencho; 1, 341b4_F/806_R; 2, N341b4_F/806_R; 3, Pro341F/Pro805R; EKW, East Kolkata wetland.

Of 254 genera recognized altogether, Figure 11 shows the distribution of the top 14 most abundant genera: *Clostridium sensu stricto*, *Psychrobacter*, *Methanothrix*, *Bacillus*, *Romboutsia*, *Planomicrobium*, *Prevotella*, *Sporacetigenium*, *Trichococcus*, *Gaiella*, *Planococcus*, *Bifidobacterium*, *Ilumatobacter*, and *Litorilinea*. The genera excluding the top 14 jointly dominated the abundance in Bhomra and EKW. *Clostridium sensu stricto* and *Psychrobacter* were the two most dominant in Bhomra; *Psychrobacter* and *Trichococcus* were the two most dominant in EKW; *Methanothrix* and *Trichococcus* were less abundant in Malencho wetland. The genus *Sporacetigenium* could not be identified in EKW.

**FIGURE 12:**
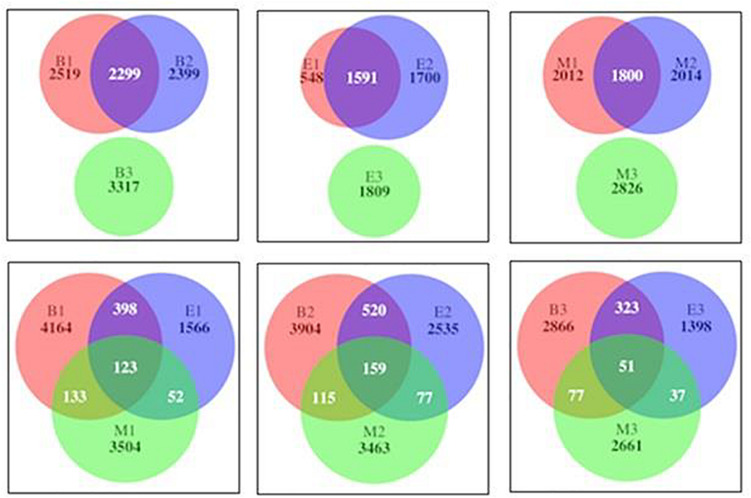
Venn diagram showing number of shared and specific prokaryote ASVs among primers within wetlands (above) and among wetlands of primers (below). The abbreviations and numerics used are as follows: B, Bhomra; E, EKW; M, Malencho; 1, 341b4_F/806_R; 2, N341b4_F/806_R; 3, Pro341F/Pro805R; ASV, amplicon sequence variant; EKW, East Kolkata wetland.

The numbers of the shared genera of primers within wetlands were 150, 83, and 178 for Bhomra, EKW, and Malencho, respectively (Supplementary Figure 2). The same for wetland among primers were 104, 131, and 58 for primers 341b4_F/806_R, N341b4_F/806_R, and Pro341F/Pro805R, respectively. As for ASV sharing, a few ASVs—i.e., 123 (1.24%), 159 (1.47%), and 51 (0.68%)—were shared by primers 341b4_F/806_R, N341b4_F/806_R, and Pro341F/Pro805R, respectively, when pooled over wetlands. Two primers—341b4_F/806_R and N341b4_F/806_R—shared 2,299 (21%), 1,591 (28%), and 1,800 (20.8%) ASVs within Bhomra, EKW, and Malencho, respectively. ASV detection capability of the primer— Pro341F/Pro805R—was completely distinct (Figure 12) from that of the other two primers in wetlands. The total ASVs—when pooled over wetlands—identified were more for newly developed primer (N341b4_F/806_R) than for the other two.

### Abode Preference and Ecosystem-Specific Indicator Microbial Taxa

The indicator analysis based on correlation index identified 11 phyla as an indicator for wetland abode preference (Table 2). Acetothermia has a high positive index value (= 0.944), indicating a tendency to live in EKW, whereas it avoids staying in Bhomra and Malencho, as it has moderate negative values. Actinobacteria and Tenericutes preferred Malencho to Bhomra and EKW as their abode. The other phyla, *viz*., Aminicenantes, Chlorobi, Crenarchaeota, Euryarchaeota, Spirochaetes, and Thaumarchaeota, preferred Bhomra as their abode.

**TABLE 2:** Phyla correlation index values and IndVal of different wetlands.

Sl. no.	Phyla	Bhomra	EKW	Malencho	IndVal	*p*-value
				
		Correlation index values				
1.	Acetothermia	−0.472	0.944	−0.472	1.000	0.041
2.	Actinobacteria	−0.468	−0.49	0.958	0.721	0.035
3.	Aminicenantes	0.945	−0.267	−0.678	0.868	0.033
4.	Chlorobi	0.955	−0.478	−0.478	1.000	0.033
5.	Crenarchaeota	0.967	−0.390	−0.577	0.928	0.033
6.	Deinococcus–Thermus	−0.381	−0.408	0.789	0.938	0.035
7.	Euryarchaeota	0.889	−0.229	−0.66	0.877	0.033
8.	Spirochaetes	0.816	−0.741	−0.075	0.745	0.033
9.	Tenericutes	−0.503	−0.489	0.992	0.993	0.035
10.	Thaumarchaeota	0.783	−0.434	−0.349	0.955	0.033
11.	Woesearchaeota	−0.523	−0.216	0.738	0.843	0.035

*The correlation indices determined a taxon’s abode preference, and the IndVal (p < 0.05) identified diagnostic or bioindicator taxon for a particular wetland. EKW, East Kolkata wetland.*

The threshold cutoff value of 0.90 for both A and B revealed diagnostic phyla for three wetlands, as follows: Chlorobi and Thaumarchaeota for Bhomra; Acetothermia for EKW; and Tenericutes for Malencho (*p*-values < 0.05). Further, of 989 second-order phyla, 173 were statistically significant indicators (Supplementary Table 5).

For the genus, the indicator analysis indicated statistical significance (*p*-value < 0.05) in 142 indicator genera (Supplementary Table 6). Among those, 38, 10, and 43 genera were more associated with Bhomra, EKW, and Malencho, respectively, at a threshold correlation index > 0.90. Setting the value of both A and B as 1.00 means that the genus is detectable as well as predictable with certainty; the analysis showed 21, 4, and 29 genera as the diagnostic genera for Bhomra, EKW, and Malencho, respectively (Supplementary Table 6). Most of the diagnostic genera had sequence similarities (NCBI BLAST) greater than 97% (Table 3) with their closest relative (at species level).

**TABLE 3:** Diagnostic genera and their closest relative (at species level).

Sl. No	Diagnostic genera	Closest relative at species level (GenBank accession number)	Identity%	*e*-Value
	**Bhomra**			
1.	*Actinocorallia*	*Actinocorallia aurantiaca* (MN199511.1)	99.01	0
2.	*Blastochloris*	*Blastochloris sulfoviridis* (NR_037121.1)	100	0
3.	*Blastococcus*	*Blastococcus aggregatus* (MK696405.1)	100	0
4.	*Bradyrhizobium*	*Bradyrhizobium elkanii* (MT426001.1)	100	0
5.	*Chlorobaculum*	*Chlorobaculum limnaeum* (NR_104865.1)	96.93	0
6.	*Chlorobium*	*Chlorobium phaeobacteroides* (NR_074352.1)	95.73	0
7.	*Lacibacterium*	*Lacibacterium aquatile* (NR_125556.1)	94.57	7.00E−179
8.	*Luteimonas*	*Luteimonas marina* (NR_044458.1)	99.77	0
9.	*Methylocaldum*	*Methylocaldum tepidum* (NR_026062.1)	99.53	0
10.	*Myxococcus*	*Myxococcus macrosporus* (NR_118593.1)	99.53	0
11.	*Non-omuraea*	*Non-omuraea harbinensis* (NR_125658.1)	100	0
12.	*Phenylobacterium*	*Phenylobacterium immobile* (NR_026498.1)	99.5	0
13.	*Phycicoccus*	*Phycicoccus dokdonensis* (NR_044286.1)	99.76	0
14.	*Pseudoxanthomonas*	*Pseudoxanthomonas mexicana* (NR_025105.1)	100	0
15.	*Rummeliibacillus*	*Rummeliibacillus pycnus* (NR_041521.1)	100	0
16.	*Saccharomonospora*	*Saccharomonospora xinjiangensis* (NR_042059.1)	99.51	0
17.	*Serpens*	*Pseudomonas flexibilis* (NR_043990.1)	100	0
18.	*Solirubrobacter*	*Solirubrobacter ginsenosidimutans* (NR_108192.1)	96.97	0
19.	*Sorangium*	*Sorangium cellulosum* (NR_116678.1)	99.07	0
20.	*Sphingopyxis*	*Sphingopyxis soli* (NR_116739.1)	99.75	0
21.	*Syntrophus*	*Syntrophus aciditrophicus* (NR_102776.1)	99.3	0
	**EKW**			
22.	*Brevinema*	*Brevinema andersonii* (NR_104855.1)	84.65	8.00E−119
23.	*Lactococcus*	*Lactococcus chungangensis* (NR_044357.1)	99.77	0
24.	*Proteocatella*	*Proteocatella sphenisci* (NR_041885.1)	98.01	0
25.	*Thermomonas*	*Thermomonas haemolytica* (NR_025441.1)	97.67	0
	**Malencho**			
26.	*Aciditerrimonas*	None		
27.	*Anderseniella*	*Anderseniella baltica* (NR_042626.1)	98.02	0
28.	*Aquiflexum*	*Aquiflexum balticum* (NR_025634.1)	99.76	0
29.	*Azoarcus*	*Azoarcus toluclasticus* (KJ000885.1)	98.84	0
30.	*Caldimonas*	*Caldimonas manganoxidans* (KY060005.1)	97.2	0
31.	*Cellvibrio*	*Cellvibrio diazotrophicus* (NR_133718.1)	96.5	0
32.	*Desulfocapsa*	None		
33.	*Desulfopila*	*Desulfobacterium corrodens* (AY274450.1)	96.99	0
34.	*Desulfosarcina*	*Desulfosarcina variabilis* (M34407.1)	98.6	0
35.	*Erythrobacter*	*Erythrobacter vulgaris* (MN746260.1)	99.75	0
36.	*Eubacterium*	*Eubacterium sulci* (NR_025289.1)	98.52	0
37.	*Filomicrobium*	None		
38.	*Haliea*	*Haliea salexigens* (NR_042993.1)	95.35	0
39.	*Klebsiella*	*Klebsiella pneumoniae* (MT439081.1)	100%	0
40.	*Mariniradius*	*Mariniradius saccharolyticus* (NR_117078.1)	98.58	0
41.	*Marinobacter*	*Marinobacter koreensis* (HE964770.1)	99.53	0
42.	*Microbulbifer*	*Microbulbifer aggregans* (NR_158143.1)	98.6	0
43.	*Nitriliruptor*	None		
44.	*Oceanirhabdus*	None		
45.	*Paenisporosarcina*	*Paenisporosarcina quisquiliarum* (MK484587.1)	100	0
46.	*Prosthecobacter*	None		
47.	*Rhodovulum*	*Limibaculum halophilum* (MT112381.1)	97.52%	0
48.	*Rubinisphaera*	None		
49.	*Sandarakinorhabdus*	*Sandarakinorhabdus cyanobacteriorum* (NR_159915.1)	98.51	0
50.	*Shivajiella*	*Shivajiella indica* (NR_117056.1)	100	0
51.	*Sporosalibacterium*	*Sporosalibacterium tautonense* (NR_156896.1)	97.28	0
52.	*Tetrasphaera*	*Phycicoccus endophyticus* (NR_148775.1)	99.51	0
53.	*Thioprofundum*	None		
54.	*Vulcanibacillus*	*Vulcanibacillus modesticaldus* (NR_042421.1)	98.14	0

*The diagnostic genera for a particular wetland were identified by setting IndVal threshold: 0.90 (p-values < 0.05). NCBI BLAST 16S rRNA database search identified closest relative at species level. The identity percentage and e-values are derived from NCBI BLAST search. No significant similarity of sequence to database is indicated as none. EKW, East Kolkata wetland; NCBI, National Center for Biotechnology Information; BLAST, Basic Local Alignment Search Tool.*

## Discussion

### Richness and Diversity of Microbial Taxa

Wetlands are recognized as a hot spot for studying microbial diversity and their role in ecology. However, research investigating microbial communities in different types of wetlands has seldom been reported (Gutknecht et al., 2006; Iliev et al., 2019; Wang et al., 2020). The microbial richness, evenness, and diversity are very important early indicators to predict the possible variation in community composition and the systems functionalities (Santos et al., 2019). In the present study, we compared the richness and diversity indices of microbial ASVs obtained from next-generation sequencing (NGS) following the same experimental conditions as suggested by Urakawa et al. (2017), to avoid biases associated with target amplification and bioinformatics analysis (Huse et al., 2010; Wang et al., 2012; Thijs et al., 2017).

The saturation of rarefaction curve in the present study around 70,000 sequence reads indicates that the probability of encountering a new ASV beyond 70,000 reads is almost negligible. Bhomra showed the steepest slope of the rarefaction curve among the wetlands, indicating higher chances of ASV detection rate followed by Malencho and EKW. Similarly, primer combination 341b4_F/806_R showed the steepest slope in Bhomra and Malencho. Therefore, the ASV detection rate is higher by primer 341b4_F/806_R than other primers in these two wetlands. The EKW revealed a noticeably different pattern of ASVs, wherein the detection rate of primer N341b4_F/806_R is the highest. This indicates the better performance of primer N341b4_F/806_R for a sewage-fed wetland. Further, the estimated expected number of ASVs for all samples suggested that the primer 341b4_F/806_R has the highest ASV detection capability followed by N341b4_F/806_R and Pro341F/Pro805R within Bhomra and Malencho wetlands. On the contrary, primer N341b4_F/806_R has the highest ASV detection capability followed by 341b4_F/806_R and Pro341F/Pro805R within EKW.

The “*k-dominance*” curve indicates more representation of certain taxa by primer Pro341F/Pro805R and less coverage of all other taxa in three wetlands, which is in agreement with Thijs et al. (2017). A similar pattern was also evident from the lowest observed and expected numbers of ASVs identified by the primer for all the wetlands. Contrary to the richness, Shannon’s diversity index is much less sensitive to sequencing and PCR errors (Kunin et al., 2010; Wu et al., 2010; Zinger et al., 2011). The Shannon indices of the three types of sediments determined in the present study (6.3 to 6.9) were comparable with those recently reported using pyrosequencing methods, even though different methodologies were employed (Thijs et al., 2017; Iliev et al., 2019; Wang et al., 2020).

Prokaryote community composition (weighted UniFrac distance matrix) was similar between primers but differed between wetlands. Thijs et al. (2017) found the bulk soil and rhizosphere soil clustered with high concordance based on weighted UniFrac distance matrix per primer pairs. However, Iliev et al. (2019) showed significant differences in the taxa composition between a natural wetland and rice paddies based on MANOVA and ANOSIM. Our result based on weighted UniFrac is also supported by ANOSIM analysis.

The present study results revealed the highest prokaryote richness and diversity in the freshwater wetland (Bhomra), followed by the brackish-water bheri (Malencho) and the sewage-fed wetland (EKW). For richness, and the number of observed and expected ASVs, the three wetlands behaved distinctly. Higher richness values were observed in two natural wetlands, Bhomra and Malencho, and very low richness value was observed in sewage-fed wetland. Further, the Shannon diversity indices differentiated Bhomra in one group and Malencho and EKW in another group. The Simpson index further differentiates the diversity between EKW and Malencho. The highest microbial diversity in Bhomra wetland may be attributed to nutrient-rich environment and more availability of organic matter in sediment due to decay of aquatic macrophytes present in the system (Decamp and Warren, 2001; Urakawa and Bernhard, 2017). The higher soil organic carbon in Bhomra due to the decay of macrophytes provides buffering effects on the environment (Marschner et al., 2003), which supports higher microbial diversity. The lower prokaryote diversity in Malencho compared with Bhomra is also consistent with recent meta-analysis results—higher levels of diversity of both bacterial and archaeal communities in inland freshwater than those of saline waters (Auguet et al., 2010; Barberán and Casamayor, 2010; Wang et al., 2012). The low prokaryote diversity in EKW might be due to sewage, which decreased the bacterial and archaeal diversity (Gu et al., 2018). It has also been reported that compared with natural wetlands, constructed wetlands used to treat city effluents have lower microbial diversity with higher abundance and functions (Hartman et al., 2008; Urakawa et al., 2017). Furthermore, the higher microbial diversity signals pristine, healthy, and balanced microbial community—the low microbial diversity is not desirable, implicating distressed communities in terms of ecosystem functioning (Bell et al., 2005; Delgado-Baquerizo et al., 2016). The Bhomra (with the highest diversity) is a less perturbed wetland than the sewage-fed EKW (with the lowest diversity).

### Preferential Wetland Abode Suitability of Phyla and Genera

The dominant prokaryote communities detected in wetland sediments include Proteobacteria, Firmicutes, Acidobacteria, Euryarchaeota, Actinobacteria, Chloroflexi, Bacteroidetes, Aminicenantes, Planctomycetes, Crenarchaeota, Cyanobacteria, and Verrucomicrobia. Many other studies also reported Proteobacteria, Bacteroidetes, Firmicutes, Acidobacteria, and Actinobacteria as dominant phyla in sediment (Hartman et al., 2008; Ligi et al., 2014; Lv et al., 2014; Sánchez, 2017), whereas Chloroflexi, Planctomycetes, Cyanobacteria, and Verrucomicrobia contribute a large fraction of microbial communities occasionally (Dorador et al., 2013; Peralta et al., 2013). We observed Firmicutes as the second dominant taxon (24.47–26.72%) in Bhomra and Malencho, whereas Acidobacteria (11.55%) in EKW. However, Lv et al. (2014) identified Bacteroidetes as the second dominant taxa (Lv et al., 2014). The microbes of these groups play a distinct role in the carbon cycle; they are capable of degrading polysaccharides—including cellulose and hemicellulose (Chapman et al., 2017; Speirs et al., 2019)—and have been identified in various habitats worldwide.

Taxonomic classification of bacteria and archaea at a higher rank, for example, phylum level, exhibits ecological coherence; and their community composition may be a valuable indicator in predicting ecosystem function (Philippot et al., 2010; Gu et al., 2018). Our results evidently reflected it: the wetland revealed a distinct pattern of the microbial community composition at the phylum level. Similar to other studies (Liu et al., 2014; Fang et al., 2015; Sun et al., 2019), the Proteobacteria dominates the community, which might be due to its diverse role in the transformation of major compounds like organic matter, nitrogen, and sulfur (Wang et al., 2016; Zheng et al., 2018). Also, the higher abundance of Proteobacteria in EKW might be due to the availability of more biodegradable organic pollutants (Cheng et al., 2014; Liu et al., 2014; Zhang et al., 2014). Apart from Proteobacteria, we found Acidobacteria, Chloroflexi, Planctomycetes, and Cyanobacteria in the EKW, similar to the findings of Torres et al. (2019) in the polluted mangrove ecosystem. It indicates that pollutants might regulate these microbial communities. Many members of these phyla have also been found to play a vital role in some processes like sewage remediation by ammonia oxidation (Wiegand et al., 2020), metal-ion-contaminated soil recovery by detoxification (Jiang et al., 2013; Torres et al., 2019), organic matter (Yamada et al., 2005; Kawaichi et al., 2013; Torres et al., 2019) and xenobiotic compound (Huang et al., 2016; Cao et al., 2017; Torres et al., 2019) decomposition and eutrophication. We also discovered a higher abundance of Verrucomicrobia in EKW, which could be attributed to high nutrient availability (He et al., 2017).

Higher abundance of saprophytic microbes—e.g., Bacteroidetes, Firmicutes, and Actinobacteria—in the sediment of the brackish-water wetland than freshwater is comparable with sediment microbial diversity of Pearl river wetland (Wang et al., 2012). These bacterial groups contain the majority of the genes encoding enzymes involved in the degradation of the soluble organic compound for the marine carbon cycle (Chapman et al., 2017), particularly those involved in the degradation of high-molecular-weight compounds such as cellulose, agar, chitin, chitosan, and amino polysaccharides (Dorador et al., 2009; Chater, 2016; Lacombe-Harvey et al., 2018). We also detected the second-order indicator phyla combinations; however, their ecological significance has yet to be determined.

In the Bhomra wetland, the archaea (Euryarchaeota and Crenarchaeota) and bacteria Aminicenantes formed the dominant microbiota. Inconsistent results available on the major archaeal groups present in wetland ecosystems (Li et al., 2017). A metadata analysis of prokaryote diversity revealed Euryarchaeota and Crenarchaeota as two major phyla in wetlands with a more relative abundance of Euryarchaeota (Lv et al., 2014)—similar to the present finding. The higher abundance of archaea in Bhomra might be due to the presence of diverse aquatic vegetation (Li et al., 2017), higher soluble organic matter (Steinmuller and Chambers, 2019), and less pollutants (Wei et al., 2019). Aminicenantes represent poorly characterized bacterial lineage in various ecosystems. They are most abundant in the freshwater environment and least abundant in heavy metal-polluted and hydrocarbon impacted environments (Farag et al., 2014; Bai et al., 2017), which might be the reason for their higher abundance in the Bhomra wetland.

Microbial communities in sediment differ depending on the types of wetlands and the anthropogenic loads from its catchment, indicating their potential use in monitoring wetland ecosystems (Urakawa and Bernhard, 2017). The present study has identified 11 phyla as putative indicators of different wetland: Acetothermia prefer EKW; four phyla (i.e., Actinobacteria, Deinococcus–Thermus, Tenericutes, and Woesearchaeota) prefer Malencho wetland; and six phyla (i.e., Aminicenantes, Chlorobi, Crenarchaeota, Euryarchaeota, Spirochaetes, and Thaumarchaeota) prefer Bhomra as their abode. Actinobacteria, Aminicenantes, Crenarchaeota, and Euryarchaeota are also dominant communities in their respective wetlands. Other phyla represent minor microbial communities—rare taxa may serve as important microbial indicators (Sogin et al., 2006; Rodriguez-Sanchez et al., 2016; Urakawa et al., 2017) for the ecosystem.

More knowledge of microbial populations through genus level ecotype identification is needed to use them as better indicators in wetland management (Martens-Habbena et al., 2009; Hernández et al., 2015; Urakawa et al., 2017). In the present study, we identified 54 diagnostic genera having differential abode preference for Bhomra, EKW, and Malencho wetlands. Close relatives for many of the ASVs of the diagnostic genera were distinct and significantly different in each wetland ecosystem, yet some ASVs remained unidentified. This indicates that these ASVs are likely to be a potential novel species (Zhao et al., 2018). Furthermore, identification of the abode preference of microorganisms based on the 16S rRNA gene (physiological capacities of bacteria) can provide significant insights into the potential roles that they play in ecological functioning of the wetlands (Zhao et al., 2018).

Various aerobic and anaerobic microorganisms derive their energy from the terminal process of organic matter mineralization by respiration using different electron acceptors available in the wetland sediments. However, the availability of different electron acceptors may depend on the metabolism of decomposers and fermenters that produce simpler organic compounds (Bongoua-Devisme et al., 2013). In the present study, we found primary decomposers and fermenters, such as a putative carbohydrate-fermenting bacterium *Hydrogenispora* in Bhomra and *Dorea* in Malencho and the putative cellulose-decomposing microorganism of genus *Sorangium* in Bhomra and genus *Cellulosilyticum* in Malencho. The secondary fermenters observed are a putative lactate-oxidizing bacterium *Serpens* in Bhomra; *Lactococcus* and an anaerobic propionate-degrading syntroph *Smithella* in EKW; and a hydrogen-oxidizing bacterium *Hydrogenophaga* in Malencho. These secondary metabolites are used by sulfate reducer and methanogens (Plugge et al., 2011). Further, the availability of these electron acceptors and their sequential depletion with an increase of depth in the sediment vertically provide thermodynamic constraints on respiration (Froelich et al., 1979). Electron acceptors, which provide higher energy yields, such as oxygen, nitrate, Mn(IV), and Fe(III), prevail near the sediment surface, followed by sulfate reducers and methanogens (Jørgensen et al., 2019b).

When oxygen is depleted at a depth of usually 3–10 cm, nitrate, Mn oxides, and Fe oxides are the next most important oxidants for organic matter. The present study has also encountered a putative Mn-oxidizing bacterium *Caldimonas manganoxidans*, an Fe(II)-oxidizing microorganism *Sideroxydans*, Fe-reducing microorganisms *Aciditerrimonas* and *Anaerosolibacter*, and nitrate-reducing bacteria *Shivajiella indica* and *Vulcanibacillus modesticaldus* in brackish-water bheri. In addition to oxidizing organic matter, these microbes are also oxidants for a significant fraction of the sulfide (Jørgensen, 1982; Canfield et al., 1992; Jørgensen and Nelson, 2004; Pellerin et al., 2015; Jørgensen et al., 2019a) produced by sulfate reducer.

In the marine sediments, dissimilatory sulfate reduction to sulfide by anaerobic microorganisms (Jørgensen and Kasten, 2006) may account for half of the organic carbon mineralization (Jørgensen, 1982; Jørgensen et al., 2019b). In the present study, the presence of diverse sulfate reducers (*Desulfocapsa*, *Desulfopila*, *Desulfosarcina*, *Desulfosporosinus*, *Desulfobulbus*, and *Desulfuromonas*) in the brackish-water bheri, Malencho, might indicate their association with sulfate reduction. Although documented primarily in salt or brackish marshes, recently, their occurrence is evident even in freshwater wetlands and peatlands, where a relatively small population of sulfate-reducing bacteria (SRB) represent an important fate for C (Pester et al., 2012; Hausmann et al., 2016; Kearns et al., 2016). In the present analysis, we noticed abode preference for green sulfur bacteria (*Chlorobaculum limnaeum* and *Chlorobium phaeobacteroides*) and purple photosynthetic bacteria (*Blastochloris sulfoviridis*) in Bhomra. These obligate photolithoautotrophs perform anaerobic photosynthesis with oxidation of inorganic sulfur compounds such as sulfide, polysulfide, or thiosulfate (Gregersen et al., 2011; Madigan et al., 2015). Besides, our study also has detected abode preference of sulfate reducers and an acetate-degrading bacterium (*Desulfobacca*) and SRB oxidizing aniline and 4-chlorophenol (*Desulfomonile* and *Desulfatiglans*) in EKW. In addition, sulfur-oxidizing bacteria such as *Thioprofundum*, *Sulfurovum*, and *Thiobacillus* are also encountered in Malencho. At present, little information is available about sulfur-oxidizing bacteria. However, it drives the oxidization of sulfur compounds produced by the activity of SRB and other sulfur compounds to sulfuric acid (Okabe et al., 2007; Tian et al., 2017).

Methanogens play an important role in the decay of organic matter in an anaerobic ecosystem and act as a terminal process of organic matter mineralization in sulfate-depleted zone (Jørgensen et al., 2019a). The present study has not found the abode preference of methanogenic archaea in Malencho. This might be due to rapid re-oxidation of the produced sulfide, more availability of sulfate, and less availability of organic C content in coastal sediment (Canfield et al., 1993; Egger et al., 2018; Jørgensen et al., 2019b). In freshwater also, methanogenesis is restricted to sulfate-depleted zone. The present study has recognized abode preference of different methanogens of the genera *Methanosarcina*, *Methanoregula*, *Methanobacterium*, *Methanocella*, and *Methanomassiliicoccus* and methanotrophs *Methylocaldum*, *Methanothrix*, and *Methylobacter* in Bhomra wetland, which indicates that methanogen bacteria preferred freshwater wetlands. It might be due to the depletion of sulfate with depth, presence of high organic carbon, and low sulfate content in freshwater sediment (Pester et al., 2012). The methanotrophs *Methylocaldum*, *Methanothrix*, and *Methylobacter* have also been observed in Bhomra. We also recorded abode preference of methanogen *Methanomethylovorans* and methanotrophs *Methylosarcina* and *Methylocystis* in EKW. This indicates that salinity could expand the abundance and diversity of SRB and decrease the diversity of methanogenic archaea and subsequently the methane emission from soil (Kroeger et al., 2017; Urakawa and Bernhard, 2017). These microbial populations could be good microbial indicators to assess the impacts of salinity.

Further, in respect to the nitrogen cycle, we found abode preference of many microbes such as *Cellvibrio diazotrophicus*, a nitrogen-fixing bacterium; *Azoarcus toluclasticus*, a denitrifying bacterium associated with nitrogen transformation that degrade aromatic compounds; and nitrate-reducing microbes *S. indica* and *V. modesticaldus* in Malencho. In Bhomra, we found abode preference for nitrous oxide-producing microbes *Luteimonas marina* and *Pseudoxanthomonas mexicana* and a nitrogen-fixing bacterium *Bradyrhizobium elkanii*, which are associated with root nodule. Besides these bacteria, we also found rhizosphere-associated bacteria: *Non-omuraea* in Bhomra, *Sideroxydans* and *Tetrasphaera* in Malencho, and an endophytic bacterium *Phycicoccus endophyticus* in Malencho.

Additionally, many of the spore-forming microorganisms, such as *Actinocorallia aurantiaca*, *Rummeliibacillus pycnus*, *Saccharomonospora xinjiangensis*, *Sporomusa*, *Micromonospora*, *Sporacetigenium*, *Paenibacillus*, and *Anaerobacter*, showed abode preference for Bhomra; a few microorganisms, e.g., *Proteocatella sphenisci*, showed abode preference for EKW; and *Desulfosporosinus* showed abode preference for Malencho. This result signifies spore formation as an alternate strategy for the survival of these microorganisms in the wetland environment. We also found microorganisms that are thermotolerant or halophilic or found from hydrothermal areas such as *Rubinisphaera*, *Sporosalibacterium*, *Thioprofundum*, *Vulcanibacillus*, *Roseiflexus*, and *Thermoleophilum*; radiation-resistant genus *Truepera*; and air-loving microorganism *Litorilinea aerophila* in Malencho. This abode preference in Malencho might be due to the less depth of ecosystem as well as periodical drying of the ecosystem.

Distinct microorganism in each wetland ecosystem has been found with their closest relative in the ecosystem having a similar environmental condition. We found microorganisms such as *Defluviicoccus*, *Lactococcus chungangensis*, *Rhodoligotrophos*, and *Trichococcus* in EKW, whose closest relatives were inhabitants of sewage/sludge; and *Anaerovorax*, a putrescine-fermenting, strictly anaerobic bacterium associated with the a foul odor in Malencho. Similarly, we found haloalkaliphilic/halophilic microorganism (*Nitriliruptor* and *Haloplasma*), a bacterium liking alkaline environments (*Alkaliphilus*), and microorganisms that inhabit sea sediment/seawater/saline water/brackish-water/estuary/mangrove (*Ilumatobacter*, *Anderseniella baltica*, *Aquiflexum balticum*, *Filomicrobium*, *Haliea salexigens*, *Mariniradius saccharolyticus*, *Marinobacter koreensis*, *Halobacillus*, *Microbulbifer aggregans*, *Oceanirhabdus sediminicola*, and *Haliangium*) in Malencho. In Bhomra, we found freshwater inhabitants (*Aquihabitans*). In Bhomra, we also found abode preference of microorganisms inhabiting forest soil (*Parasegetibacter*), which might be due to the presence of organic matter derived from diverse aquatic vegetation. The abode preference of these microorganism signifies salinity along with sewage, playing a vital role in shaping microbial communities. Previous studies have also found salinity and sewage (Hu et al., 2014; Chambers et al., 2016; Wei et al., 2019) as important factors for distinct microbial community structure.

Microorganisms *Eubacterium sulci*, *Stomatobaculum*, *Coprococcus*, *Roseburia*, *Paraprevotella*, and *Dorea* in Malencho; *Cetobacterium* in EKW; and *Aerococcus* in Bhomra associated with human urine, pig urine, vagina, feces, and intestine were observed. Microorganisms such as *Succinivibrio* and *Blautia* from Malencho, *Proteocatella* from EKW, and *Erythrobacter vulgaris* and *Symbiobacterium* from Malencho, associated with other animal source and marine invertebrates, were also found. The putative genus of the infectious microorganism of animal, *Brevinema*, and disease-causing microorganism *Clostridium_III* and *Klebsiella pneumoniae* were observed in EKW, Bhomra, and Malencho, respectively. Further, *Thermomonas*, a blood-dissolving microorganism, in EKW and *Turicibacter* in Malencho wetland were observed. As the wetlands are closely located in human habitation, the release of pathogens associated with different secretions and excretions of human, animal, and fish is inevitable. Further, it indicates wetlands are conducive enough to act as habitats for these microorganisms (Wu et al., 2016); however, concern on their public health aspects can only be ascertained by their seasonal abundance pattern in the wetlands and their transfer to human and animals through fish and other edible components and drinking water.

## Conclusion

This study presents the first detailed and simultaneous characterization of the bacterial and archaeal communities and the identification of their abode in the freshwater wetland (Bhomra), brackish-water bheri (Malencho), and sewage-fed wetland (EKW) sediment, using three universal primers. It revealed that α-diversity indices and phylogenetic differential abundance pattern (weighted UniFrac) did not differ significantly among primers; however, those were significantly different across wetlands. Further, ASV similarity via Venn diagram analysis showed that primer pairs 341b4_F/806_R and N341b4_F/806_R shared 20.8–28% ASVs within the wetland, whereas primer pair Pro341F/Pro805R was completely distinct. More numbers of ASVs have been identified by the newly developed primer combination (N341b4_F/806_R), opening further useful scope for the study of diverse ecosystems. Overall, a distinct diversity pattern differentiates freshwater wetland, brackish-water bheri, and sewage-fed wetland ecosystems. Comparison of prokaryote communities in three different wetland sediments revealed the highest prokaryote richness and diversity in freshwater wetland, followed by brackish-water bheri and sewage-fed wetland. The identification of closest relative (at species level) of the diagnostic genus revealed the abode preference of prokaryotes such as sulfate reducers in Malencho, methanogens in Bhomra, and sewage-loving microbes in EKW. The phylogenetic differential abundance pattern along with distinct abode preference of these microorganisms signifies salinity along with sewage and plays a vital role in shaping microbial communities. Such information is essential to better understand the microbial ecology of wetlands.

## Data Availability Statement

Raw read data are available at the National Center for Biotechnology Information Sequence Read Archive (Bio project Number PRJNA492841). Prokaryote 16S rRNA sequences are available on GenBank (accession number SAMN10107905 - SAMN10107913).

## Author Contributions

KK did the conceptualization, methodology, investigation, sample collection, data curation, laboratory analysis, bioinformatics, writing—original draft, and writing—review and editing. MN did the bioinformatics and statistical analysis, writing—original draft, and writing—review and editing. MA wrote—review and edited. SD did the sample collection, manuscript review, and editing. BG did the preparation of location map and analysis of water and sediment quality parameters. US did the supervision and overall guidance. SN did the analysis of water and sediment quality parameters. CJ did the statistical computation. BD did the guidance for manuscript preparation. All authors contributed to the article and approved the submitted version.

## Conflict of Interest

The authors declare that the research was conducted in the absence of any commercial or financial relationships that could be construed as a potential conflict of interest.

## Supplementary Material

Additional information on primers detail, raw and processed reads data statistics, primer specific taxa abundance, phylum and genus level analysis has been given in Supplementary information section.

## Abbreviations

ANOSIM: analysis of Similarities
ANOVA: analysis of Variance
ASVs: amplicon sequence variants
bp: base pair
DNA: deoxyribonucleic acid
EKW: East Kolkata Wetland
MANOVA: multivariate analysis of variance
PCR: polymerase chain reaction
SRB: sulfate-reducing bacteria
TIC: total inorganic carbon
TOC: total organic carbon.
