# Supplementary material for: Evaluation of Three Prokaryote Primers for Identification of Prokaryote Community Structure and Their Abode Preference in Three Distinct Wetland Ecosystems
Source: Front Microbiol. 2021 Jul 14;12:643945. doi: 10.3389/fmicb.2021.643945 (PMC8317468; doi:10.3389/fmicb.2021.643945)
Supplement: Supplementary file 1 [file Data_Sheet_1.pdf]

Supplementary Table 1 Details about primers used.

Sl. No.	Primers	5’-3’	References
1.	341b4_F	CTAYGGRRBGCWGCAG	Lu et. al., 2015
	806_R	GGACTACNNGGGTATCTAAT	
2.	N341b4_F	CTAYGGRNBGCWGCAG	This study
	806_R	GGACTACNNGGGTATCTAAT	Lu et. al., 2015
3.	Pro341F	CCTACGGGNBGCASCAG	Takahashi et. al., 2014
	Pro805R	GACTACNVGGGTATCTAATCC	

Supplementary Table 2 Data statistics of Illumina MiSeq prokaryote16S rRNA amplicon raw and processed reads.

Sampling sites	Mean of library fragment size (bp)	Raw reads	Retained reads after processing
EKW-1	587	262173	110346
EKW-2	590	461999	197867
EKW-3	589	397679	95404
Bhomra-1	590	354459	139492
Bhomra-2	588	346826	132783
Bhomra-3	577	401722	93436
Malencho-1	595	404593	186216
Malencho-2	594	456255	209785
Malencho-3	583	416763	112025

Supplementary Table 3. Estimated expected number of ASVs identified by three prokaryote primers in each wetland (Bhomra, EKW, and Malencho).

**Primers**	**Bhomra**	**EKW**	**Malencho**
341b4_F/806_R	4719± 9	2121± 4	3664 ± 11
N341b4_F/806_R	4614 ± 9	3125± 11	3637 ± 12
Pro341F/Pro805R	3294 ± 5	1797 ± 4	2790 ± 6

Supplementary Table 4 Higher (> 1 %) and lower (< 1 %) abundant phyla diagnosed by three primers in each wetland; The numeric in table indicates as follows: 1= 341b4_F/806_R; 2=N341b4_F/806_R; 3=Pro341F/Pro805R.

Sl. No.	Phyla (> 1 %)	BHOMRA			EKW			MALENCHO		
		1	2	3	1	2	3	1	2	3
1.	Acidobacteria	9.85	7.62	7.46	15.63	12.55	10.37	6.65	3.99	9.07
2.	Actinobacteria	5.67	4.82	6.29	4.72	4.13	6.55	8.02	6.31	10.98
3.	Aminicenantes	3.14	2.37	3.26	0.82	0.61	0.91	0.08	0.06	0.14
4.	Bacteroidetes	4.13	5.4	0.69	2.09	2.95	0.3	9.85	11.98	0.93
5.	Chloroflexi	4.47	8.12	6.58	8.41	13.29	9.95	5.9	7.66	12.61
6.	Crenarchaeota	1.19	0.89	1.93	0.11	0.1	0.35	0.02	0.01	0.03
7.	Cyanobacteria/Chloroplast	0.99	0.81	1.37	1.66	1.34	2.82	1.4	1.23	1.57
8.	Euryarchaeota	6.69	4.91	14.27	1.82	1.44	3.6	0.05	0.05	0.13
9.	Firmicutes	26.1	24.78	23.42	9.3	10.14	9.4	28.42	28.59	22.46
10.	Planctomycetes	3.07	8.54	0.34	3.07	8.39	0.15	2.15	5.15	0.41
11.	Proteobacteria	30.4	26.65	29.85	49.6	41.16	52.06	35.08	32.15	36.89
12.	Verrucomicrobia	0.76	2.3	2.83	0.95	2.36	2.33	0.41	1.38	2.58
	**Phyla (< 1 %)**									
1.	Acetothermia	0	0	0	0.07	0.04	0.04	0	0	0
2.	Aminicenantes	3.14	2.37	3.26	0.82	0.61	0.91	0.08	0.06	0.14
3.	Armatimonadetes	0.07	0.08	0.25	0.09	0.1	0.18	0.11	0.06	0.29
4.	Bacteroidetes	4.13	5.4	0.69	2.09	2.95	0.3	9.85	11.98	0.93
5.	BRC1	0.03	0.05	0.01	0.05	0.03	0	0.03	0.01	0.01
6.	candidate_division_WPS-1	0.86	0.62	0.06	0.18	0.08	0.01	0.11	0.05	0.04
7.	Candidatus_Saccharibacteria	0.07	0.07	0.05	0.55	0.38	0.43	0.26	0.25	0.25
8.	Chlamydiae	0.15	0.18	0.01	0.12	0.09	0	0.08	0.04	0.01
9.	Chlorobi	0.49	0.32	0.4	0	0	0	0	0	0
10.	Cloacimonetes	0	0.01	0.01	0.01	0.02	0.01	0	0	0
11.	Crenarchaeota	1.19	0.89	1.93	0.11	0.1	0.35	0.02	0.01	0.03
12.	Cyanobacteria/Chloroplast	0.99	0.81	1.37	1.66	1.34	2.82	1.4	1.23	1.57
13.	Deferribacteres	0.02	0.01	0.02	0.02	0.01	0	0.02	0	0.01
14.	Deinococcus-Thermus	0.01	0.01	0	0	0.01	0	0.08	0.06	0.02
15.	Elusimicrobia	0.02	0	0	0	0	0	0.02	0	0
16.	Euryarchaeota	6.69	4.91	14.27	1.82	1.44	3.6	0.05	0.05	0.13
17.	Fusobacteria	0.03	0.03	0	0.07	0.06	0.02	0.06	0.02	0
18.	Gemmatimonadetes	0.06	0.04	0.06	0	0	0	0.05	0.05	0.05
19.	Hydrogenedentes	0.01	0.09	0.08	0	0.08	0.02	0.02	0.07	0.06
20.	Ignavibacteriae	0.04	0.02	0.01	0.13	0.1	0.03	0.21	0.15	0.11
21.	Latescibacteria	0.31	0.19	0.15	0.2	0.14	0.19	0.09	0.09	0.09
22.	Lentisphaerae	0.01	0.02	0	0.01	0	0	0.04	0.01	0.01
23.	Microgenomates	0	0	0.04	0	0	0.04	0	0	0.08

24.	Nitrospirae	0.06	0.05	0.01	0	0	0	0.01	0	0
25.	Omnitrophica	0.01	0.01	0	0	0	0	0	0	0
26.	Pacearchaeota	0.01	0.01	0.05	0.02	0.02	0.03	0.01	0.01	0.05
27.	Parcubacteria	0.14	0.12	0	0.18	0.32	0.04	0.14	0.09	0.01
28.	Planctomycetes	3.07	8.54	0.34	3.07	8.39	0.15	2.15	5.15	0.41
29.	Spirochaetes	0.22	0.14	0.34	0.04	0.02	0.11	0.08	0.06	0.15
30.	SR1	0.01	0.01	0	0	0.01	0	0	0.01	0
31.	Synergistetes	0.11	0.1	0.02	0.05	0.02	0.01	0	0	0
32.	Tenericutes	0	0	0	0.01	0	0.01	0.47	0.34	0.83
33.	Thaumarchaeota	0.81	0.61	0.12	0.01	0.01	0	0.04	0.03	0.01
34.	Verrucomicrobia	0.76	2.3	2.83	0.95	2.36	2.33	0.41	1.38	2.58
35.	Woesearchaeota	0	0	0.01	0.01	0.02	0.03	0.02	0.02	0.12

Supplementary Table 5 Second-order indicator phyla combination and its statistical significance. The IndVal (threshold: 0.90) identified diagnostic or bioindicator taxon combination for wetlands. The permutation test of IndVal index established statistical significance (p-values < 0.05) of the preferences.

Sl. No.	Phyla combination	IndVal	p-value
1.	Acetothermia	1	0.037
2.	Acetothermia+Acidobacteria	1	0.037
3.	Acetothermia+Actinobacteria	1	0.037
4.	Acetothermia+Aminicenantes	1	0.037
5.	Acetothermia+Armatimonadetes	1	0.037
6.	Acetothermia+Bacteroidetes	1	0.037
7.	Acetothermia+candidate_division_WPS-1	1	0.037
8.	Acetothermia+Candidatus_Saccharibacteria	1	0.037
9.	Acetothermia+Chlamydiae	1	0.037
10.	Acetothermia+Chloroflexi	1	0.037
11.	Acetothermia+Cloacimonetes	1	0.037
12.	Acetothermia+Crenarchaeota	1	0.037
13.	Acetothermia+Cyanobacteria/Chloroplast	1	0.037
14.	Acetothermia+Deferribacteres	1	0.037
15.	Acetothermia+Euryarchaeota	1	0.037
16.	Acetothermia+Firmicutes	1	0.037
17.	Acetothermia+Fusobacteria	1	0.037
18.	Acetothermia+Hydrogenedentes	1	0.037
19.	Acetothermia+Ignavibacteriae	1	0.037
20.	Acetothermia+Latescibacteria	1	0.037
21.	Acetothermia+Pacearchaeota	1	0.037
22.	Acetothermia+Parcubacteria	1	0.037
23.	Acetothermia+Planctomycetes	1	0.037
24.	Acetothermia+Proteobacteria	1	0.037
25.	Acetothermia+Spirochaetes	1	0.037
26.	Acetothermia+SR1	1	0.037

27.	Acetothermia+Synergistetes	1	0.037
28.	Acetothermia+Tenericutes	1	0.037
29.	Acetothermia+Verrucomicrobia	1	0.037
30.	Acetothermia+Woesearchaeota	1	0.037
31.	Acidobacteria+Chlorobi	1	0.043
32.	Acidobacteria+Cloacimonetes	1	0.047
33.	Acidobacteria+Gemmatimonadetes	1	0.037
34.	Acidobacteria+Synergistetes	1	0.047
35.	Acidobacteria+Tenericutes	0.999	0.043
36.	Actinobacteria+Chlorobi	1	0.043
37.	Actinobacteria+Cloacimonetes	1	0.047
38.	Actinobacteria+Gemmatimonadetes	1	0.037
39.	Actinobacteria+Synergistetes	1	0.047
40.	Actinobacteria+Tenericutes	0.999	0.043
41.	Aminicenantes+Chlorobi	1	0.043
42.	Aminicenantes+Cloacimonetes	1	0.047
43.	Aminicenantes+Gemmatimonadetes	1	0.037
44.	Aminicenantes+Synergistetes	1	0.047
45.	Aminicenantes+Tenericutes	0.995	0.043
46.	Armatimonadetes+Chlorobi	1	0.043
47.	Armatimonadetes+Cloacimonetes	1	0.047
48.	Armatimonadetes+Gemmatimonadetes	1	0.037
49.	Armatimonadetes+Synergistetes	1	0.047
50.	Armatimonadetes+Tenericutes	0.997	0.043
51.	Bacteroidetes+Chlorobi	1	0.043
52.	Bacteroidetes+Cloacimonetes	1	0.047
53.	Bacteroidetes+Gemmatimonadetes	1	0.037
54.	Bacteroidetes+Synergistetes	1	0.047
55.	Bacteroidetes+Tenericutes	0.999	0.043
56.	BRC1+Chlorobi	1	0.043

57.	BRC1+Gemmatimonadetes	1	0.037
58.	candidate_division_WPS-1+Chlorobi	1	0.043
59.	candidate_division_WPS-1+Cloacimonetes	1	0.047
60.	candidate_division_WPS-1+Gemmatimonadetes	1	0.037
61.	candidate_division_WPS-1+Synergistetes	1	0.047
62.	candidate_division_WPS-1+Tenericutes	0.995	0.043
63.	Candidatus_Saccharibacteria+Chlorobi	1	0.043
64.	Candidatus_Saccharibacteria+Cloacimonetes	1	0.047
65.	Candidatus_Saccharibacteria+Gemmatimonadetes	1	0.037
66.	Candidatus_Saccharibacteria+Synergistetes	1	0.047
67.	Candidatus_Saccharibacteria+Tenericutes	0.998	0.039
68.	Chlamydiae+Chlorobi	1	0.043
69.	Chlamydiae+Cloacimonetes	1	0.047
70.	Chlamydiae+Gemmatimonadetes	1	0.037
71.	Chlamydiae+Synergistetes	1	0.047
72.	Chlorobi	1	0.043
73.	Chlorobi+Chloroflexi	1	0.043
74.	Chlorobi+Cloacimonetes	1	0.043
75.	Chlorobi+Crenarchaeota	1	0.043
76.	Chlorobi+Cyanobacteria/Chloroplast	1	0.043
77.	Chlorobi+Deferribacteres	1	0.043
78.	Chlorobi+Euryarchaeota	1	0.043
79.	Chlorobi+Firmicutes	1	0.043
80.	Chlorobi+Fusobacteria	1	0.043
81.	Chlorobi+Gemmatimonadetes	1	0.043
82.	Chlorobi+Hydrogenedentes	1	0.043
83.	Chlorobi+Ignavibacteriae	1	0.043
84.	Chlorobi+Latescibacteria	1	0.043
85.	Chlorobi+Microgenomates	1	0.043
86.	Chlorobi+Nitrospirae	1	0.043

87.	Chlorobi+Pacearchaeota	1	0.043
88.	Chlorobi+Planctomycetes	1	0.043
89.	Chlorobi+Proteobacteria	1	0.043
90.	Chlorobi+Spirochaetes	1	0.043
91.	Chlorobi+Synergistetes	1	0.043
92.	Chlorobi+Thaumarchaeota	1	0.043
93.	Chlorobi+Verrucomicrobia	1	0.043
94.	Chlorobi+Woesearchaeota	1	0.043
95.	Chloroflexi+Cloacimonetes	1	0.047
96.	Chloroflexi+Gemmatimonadetes	1	0.037
97.	Chloroflexi+Synergistetes	1	0.047
98.	Chloroflexi+Tenericutes	0.999	0.043
99.	Cloacimonetes	1	0.047
100.	Cloacimonetes+Crenarchaeota	1	0.047
101.	Cloacimonetes+Cyanobacteria/Chloroplast	1	0.047
102.	Cloacimonetes+Deferribacteres	1	0.047
103.	Cloacimonetes+Euryarchaeota	1	0.047
104.	Cloacimonetes+Firmicutes	1	0.047
105.	Cloacimonetes+Fusobacteria	1	0.047
106.	Cloacimonetes+Gemmatimonadetes	1	0.043
107.	Cloacimonetes+Hydrogenedentes	1	0.047
108.	Cloacimonetes+Ignavibacteriae	1	0.047
109.	Cloacimonetes+Latescibacteria	1	0.047
110.	Cloacimonetes+Nitrospirae	1	0.043
111.	Cloacimonetes+Pacearchaeota	1	0.047
112.	Cloacimonetes+Parcubacteria	0.936	0.037
113.	Cloacimonetes+Planctomycetes	1	0.047
114.	Cloacimonetes+Proteobacteria	1	0.047
115.	Cloacimonetes+Spirochaetes	1	0.047
116.	Cloacimonetes+Synergistetes	1	0.047

117.	Cloacimonetes+Tenericutes	0.933	0.037
118.	Cloacimonetes+Verrucomicrobia	1	0.047
119.	Cloacimonetes+Woesearchaeota	1	0.047
120.	Crenarchaeota+Gemmatimonadetes	1	0.037
121.	Crenarchaeota+Synergistetes	1	0.047
122.	Crenarchaeota+Tenericutes	0.983	0.027
123.	Cyanobacteria/Chloroplast+Gemmatimonadetes	1	0.037
124.	Cyanobacteria/Chloroplast+Synergistetes	1	0.047
125.	Cyanobacteria/Chloroplast+Tenericutes	0.999	0.043
126.	Deferribacteres+Gemmatimonadetes	1	0.037
127.	Deferribacteres+Synergistetes	1	0.047
128.	Deinococcus-Thermus+Gemmatimonadetes	0.948	0.047
129.	Deinococcus-Thermus+Tenericutes	0.979	0.047
130.	Euryarchaeota+Gemmatimonadetes	1	0.037
131.	Euryarchaeota+Synergistetes	1	0.047
132.	Euryarchaeota+Tenericutes	0.994	0.043
133.	Firmicutes+Gemmatimonadetes	1	0.037
134.	Firmicutes+Synergistetes	1	0.047
135.	Firmicutes+Tenericutes	0.999	0.043
136.	Fusobacteria+Synergistetes	1	0.047
137.	Gemmatimonadetes	1	0.037
138.	Gemmatimonadetes+Hydrogenedentes	1	0.037
139.	Gemmatimonadetes+Ignavibacteriae	1	0.037
140.	Gemmatimonadetes+Latescibacteria	1	0.037
141.	Gemmatimonadetes+Pacearchaeota	1	0.037
142.	Gemmatimonadetes+Planctomycetes	1	0.037
143.	Gemmatimonadetes+Proteobacteria	1	0.037
144.	Gemmatimonadetes+Spirochaetes	1	0.037
145.	Gemmatimonadetes+Synergistetes	1	0.043
146.	Gemmatimonadetes+Tenericutes	0.991	0.047

147.	Gemmatimonadetes+Thaumarchaeota	1	0.037
148.	Gemmatimonadetes+Verrucomicrobia	1	0.037
149.	Gemmatimonadetes+Woesearchaeota	1	0.037
150.	Hydrogenedentes+Synergistetes	1	0.047
151.	Ignavibacteriae+Synergistetes	1	0.047
152.	Ignavibacteriae+Tenericutes	0.998	0.025
153.	Latescibacteria+Synergistetes	1	0.047
154.	Latescibacteria+Tenericutes	0.996	0.043
155.	Microgenomates+Nitrospirae	1	0.043
156.	Nitrospirae+Synergistetes	1	0.043
157.	Pacearchaeota+Synergistetes	1	0.047
158.	Pacearchaeota+Tenericutes	0.981	0.043
159.	Parcubacteria+Tenericutes	0.996	0.043
160.	Parcubacteria+Woesearchaeota	0.977	0.043
161.	Planctomycetes+Synergistetes	1	0.047
162.	Planctomycetes+Tenericutes	0.999	0.043
163.	Proteobacteria+Synergistetes	1	0.047
164.	Proteobacteria+Tenericutes	0.999	0.043
165.	Spirochaetes+Synergistetes	1	0.047
166.	Spirochaetes+Tenericutes	0.995	0.043
167.	Synergistetes	1	0.047
168.	Synergistetes+Tenericutes	0.928	0.037
169.	Synergistetes+Verrucomicrobia	1	0.047
170.	Synergistetes+Woesearchaeota	1	0.047
171.	Tenericutes	0.999	0.043
172.	Tenericutes+Verrucomicrobia	0.999	0.043
173.	Tenericutes+Woesearchaeota	0.994	0.043

Supplementary Table 6 Correlation Index, and IndVal at genus level. The correlation indices determined taxon's abode preference and the IndVal (threshold: 0.90) identified diagnostic or bioindicator taxon for a particular wetland. The permutation test of IndVal index established statistical significance (p-values < 0.05) of the preferences.

Sl. No.	Genera	Bhomra	EKW	Malencho	IndVal	p-value
		Correlation index values				
1.	*Aciditerrimonas*	-0.479	-0.479	0.958	1	0.044
2.	*Acinetobacter*	-0.602	0.871	-0.27	0.888	0.031
3.	*Actinocorallia*	0.956	-0.478	-0.478	1	0.037
4.	*Aerococcus*	0.984	-0.552	-0.432	0.963	0.037
5.	*Agromyces*	-0.237	-0.623	0.86	0.891	0.044
6.	*Akkermansia*	-0.393	0.824	-0.43	0.986	0.031
7.	*Alkaliphilus*	-0.481	-0.503	0.984	0.992	0.044
8.	*Altererythrobacter*	-0.436	-0.477	0.913	0.986	0.044
9.	*Anaerobacter*	0.696	-0.935	0.239	0.763	0.037
10.	*Anaeromyxobacter*	0.9	-0.41	-0.49	0.739	0.037
11.	*Anaerosolibacter*	-0.409	-0.471	0.88	0.978	0.044
12.	*Anaerovorax*	-0.192	0.848	-0.656	0.874	0.031
13.	*Anderseniella*	-0.499	-0.499	0.999	1	0.044
14.	*Aquicella*	0.856	-0.701	-0.156	0.861	0.037
15.	*Aquiflexum*	-0.492	-0.492	0.984	1	0.044
16.	*Aquihabitans*	0.935	-0.416	-0.519	0.707	0.037
17.	*Azoarcus*	-0.488	-0.488	0.977	1	0.044
18.	*Bacillus*	0.701	-0.932	0.231	0.75	0.037
19.	*Blastochloris*	0.917	-0.459	-0.459	1	0.037
20.	*Blastococcus*	0.816	-0.408	-0.408	1	0.037
21.	*Blautia*	-0.492	-0.326	0.817	0.871	0.044
22.	*Brachybacterium*	-0.274	-0.572	0.846	0.825	0.044
23.	*Bradyrhizobium*	0.968	-0.484	-0.484	1	0.037
24.	*Brevibacillus*	0.98	-0.526	-0.455	0.977	0.037
25.	*Brevinema*	-0.483	0.965	-0.483	1	0.031

26.	*Caldimonas*	-0.49	-0.49	0.981	1	0.044
27.	*Cellulosilyticum*	0.075	-0.868	0.793	0.799	0.044
28.	*Cellvibrio*	-0.481	-0.481	0.962	1	0.044
29.	*Cetobacterium*	-0.353	0.842	-0.489	0.953	0.031
30.	*Chlorobaculum*	0.943	-0.471	-0.471	1	0.037
31.	*Chlorobium*	0.94	-0.47	-0.47	1	0.037
32.	*Chondromyces*	-0.069	-0.783	0.852	0.834	0.044
33.	*Clostridium_III*	0.88	-0.69	-0.19	0.851	0.037
34.	*Clostridium_sensu_stricto*	0.947	-0.63	-0.317	0.784	0.037
35.	*Conexibacter*	-0.615	0.842	-0.227	0.837	0.031
36.	*Coprococcus*	-0.338	-0.457	0.795	0.848	0.044
37.	*Defluviicoccus*	-0.489	0.986	-0.497	0.991	0.031
38.	*Desulfatiglans*	-0.46	0.942	-0.482	0.718	0.031
39.	*Desulfobacca*	-0.41	0.883	-0.472	0.826	0.031
40.	*Desulfobulbus*	-0.368	-0.548	0.916	0.944	0.044
41.	*Desulfocapsa*	-0.491	-0.491	0.983	1	0.044
42.	*Desulfomonile*	-0.907	0.767	0.14	0.762	0.031
43.	*Desulfopila*	-0.489	-0.489	0.979	1	0.044
44.	*Desulfosarcina*	-0.494	-0.494	0.988	1	0.044
45.	*Desulfosporosinus*	-0.522	-0.468	0.99	0.983	0.044
46.	*Desulfuromonas*	-0.282	-0.659	0.94	0.889	0.044
47.	*Dorea*	-0.347	-0.506	0.852	0.833	0.044
48.	*Erythrobacter*	-0.377	-0.377	0.755	1	0.044
49.	*Eubacterium*	-0.485	-0.485	0.97	1	0.044
50.	*Fictibacillus*	0.983	-0.451	-0.532	0.964	0.037
51.	*Filomicrobium*	-0.487	-0.487	0.974	1	0.044
52.	*Fonticella*	-0.524	-0.444	0.968	0.974	0.044
53.	*Gemmata*	0.691	-0.354	-0.337	0.987	0.037
54.	*Gemmiger*	-0.4	-0.479	0.879	0.865	0.044
55.	*Haliangium*	-0.283	-0.668	0.951	0.899	0.044

56.	*Haliea*	-0.448	-0.448	0.897	1	0.044
57.	*Halobacillus*	-0.202	-0.648	0.85	0.827	0.044
58.	*Haloplasma*	-0.504	-0.487	0.991	0.994	0.044
59.	*Hydrogenispora*	0.977	-0.553	-0.424	0.904	0.037
60.	*Hydrogenophaga*	-0.683	-0.232	0.915	0.883	0.044
61.	*Ilumatobacter*	-0.483	-0.471	0.954	0.89	0.044
62.	*Klebsiella*	-0.377	-0.377	0.753	1	0.044
63.	*Lacibacterium*	0.975	-0.488	-0.488	1	0.037
64.	*Lactococcus*	-0.465	0.93	-0.465	1	0.031
65.	*Litorilinea*	-0.52	-0.406	0.926	0.863	0.044
66.	*Luteimonas*	0.86	-0.43	-0.43	1	0.037
67.	*Lysinibacillus*	0.982	-0.494	-0.488	0.99	0.037
68.	*Lysobacter*	-0.311	-0.635	0.946	0.911	0.044
69.	*Mariniradius*	-0.48	-0.48	0.96	1	0.044
70.	*Marinobacter*	-0.457	-0.457	0.913	1	0.044
71.	*Marmoricola*	-0.457	-0.519	0.976	0.974	0.044
72.	*Methanobacterium*	0.919	-0.238	-0.68	0.876	0.037
73.	*Methanocella*	0.942	-0.5	-0.442	0.945	0.037
74.	*Methanomassiliicoccus*	0.933	-0.238	-0.696	0.881	0.037
75.	*Methanomethylovorans*	-0.325	0.912	-0.588	0.923	0.031
76.	*Methanoregula*	0.989	-0.407	-0.582	0.949	0.037
77.	*Methanosarcina*	0.968	-0.437	-0.531	0.954	0.037
78.	*Methanothrix*	0.801	-0.207	-0.593	0.88	0.037
79.	*Methylobacter*	0.841	-0.701	-0.14	0.824	0.037
80.	*Methylocaldum*	0.941	-0.47	-0.47	1	0.037
81.	*Methyloceanibacter*	-0.363	-0.578	0.941	0.936	0.044
82.	*Methylocystis*	-0.163	0.916	-0.753	0.784	0.031
83.	*Methylosarcina*	-0.504	0.941	-0.437	0.83	0.031
84.	*Microbulbifer*	-0.446	-0.446	0.891	1	0.044
85.	*Micromonospora*	0.952	-0.566	-0.386	0.946	0.037

86.	*Microvirga*	0.952	-0.541	-0.411	0.931	0.037
87.	*Myxococcus*	0.988	-0.494	-0.494	1	0.037
88.	*Nitriliruptor*	-0.48	-0.48	0.96	1	0.044
89.	*Nocardioides*	0.893	-0.531	-0.362	0.933	0.037
90.	*Nonomuraea*	0.997	-0.499	-0.499	1	0.037
91.	*Oceanirhabdus*	-0.415	-0.415	0.83	1	0.044
92.	*Paenibacillus*	0.923	-0.603	-0.32	0.905	0.037
93.	*Paenisporosarcina*	-0.492	-0.492	0.984	1	0.044
94.	*Paraprevotella*	-0.399	-0.455	0.854	0.842	0.044
95.	*Parasegetibacter*	0.801	-0.471	-0.33	0.949	0.037
96.	*Pelotomaculum*	0.962	-0.481	-0.481	0.958	0.037
97.	*Phenylobacterium*	0.856	-0.428	-0.428	1	0.037
98.	*Phycicoccus*	0.978	-0.489	-0.489	1	0.037
99.	*Polyangium*	-0.339	-0.574	0.913	0.764	0.044
100.	*Prosthecobacter*	-0.346	-0.346	0.693	1	0.044
101.	*Proteocatella*	-0.422	0.845	-0.422	1	0.031
102.	*Pseudoxanthomonas*	0.984	-0.492	-0.492	1	0.037
103.	*Rhodoligotrophos*	-0.204	0.94	-0.736	0.871	0.031
104.	*Rhodovulum*	-0.487	-0.487	0.974	1	0.044
105.	*Roseburia*	-0.329	-0.496	0.825	0.846	0.044
106.	*Roseiflexus*	-0.386	-0.512	0.899	0.958	0.044
107.	*Roseomonas*	-0.34	-0.587	0.928	0.894	0.044
108.	*Rubinisphaera*	-0.36	-0.36	0.721	1	0.044
109.	*Rummeliibacillus*	0.981	-0.491	-0.491	1	0.037
110.	*Saccharomonospora*	0.981	-0.49	-0.49	1	0.037
111.	*Sandarakinorhabdus*	-0.495	-0.495	0.99	1	0.044
112.	*Selenomonas*	-0.346	-0.506	0.852	0.946	0.044
113.	*Serpens*	0.971	-0.485	-0.485	1	0.037
114.	*Shivajiella*	-0.486	-0.486	0.973	1	0.044
115.	*Sideroxydans*	-0.081	-0.73	0.811	0.839	0.044

116.	*Smithella*	-0.271	0.953	-0.682	0.894	0.031
117.	*Solirubrobacter*	0.999	-0.5	-0.5	1	0.037
118.	*Sorangium*	0.956	-0.478	-0.478	1	0.037
119.	*Sphingomonas*	0.795	-0.86	0.065	0.793	0.037
120.	*Sphingopyxis*	0.957	-0.478	-0.478	1	0.037
121.	*Sporacetigenium*	0.97	-0.597	-0.373	0.935	0.037
122.	*Sporomusa*	0.926	-0.418	-0.508	0.95	0.037
123.	*Sporosalibacterium*	-0.481	-0.481	0.962	1	0.044
124.	*Staphylococcus*	-0.383	-0.478	0.86	0.962	0.044
125.	*Steroidobacter*	-0.409	-0.558	0.967	0.954	0.044
126.	*Stomatobaculum*	-0.307	-0.43	0.737	0.951	0.044
127.	*Streptomyces*	0.961	-0.625	-0.337	0.884	0.037
128.	*Succinivibrio*	-0.334	-0.425	0.76	0.903	0.044
129.	*Sulfurovum*	-0.463	-0.484	0.947	0.993	0.044
130.	*Symbiobacterium*	-0.347	-0.607	0.954	0.926	0.044
131.	*Syntrophomonas*	0.822	-0.202	-0.619	0.87	0.037
132.	*Syntrophus*	0.941	-0.471	-0.471	1	0.037
133.	*Tetrasphaera*	-0.489	-0.489	0.979	1	0.044
134.	*Thermoleophilum*	-0.45	-0.519	0.968	0.942	0.044
135.	*Thermomonas*	-0.475	0.95	-0.475	1	0.031
136.	*Thiobacillus*	-0.467	-0.516	0.983	0.91	0.044
137.	*Thioprofundum*	-0.449	-0.449	0.898	1	0.044
138.	*Trichococcus*	-0.116	0.899	-0.783	0.846	0.031
139.	*Truepera*	-0.393	-0.407	0.8	0.964	0.044
140.	*Turicibacter*	-0.494	-0.449	0.943	0.739	0.044
141.	*Vulcanibacillus*	-0.452	-0.452	0.905	1	0.044
142.	*Zavarzinella*	0.705	-0.384	-0.322	0.965	0.037

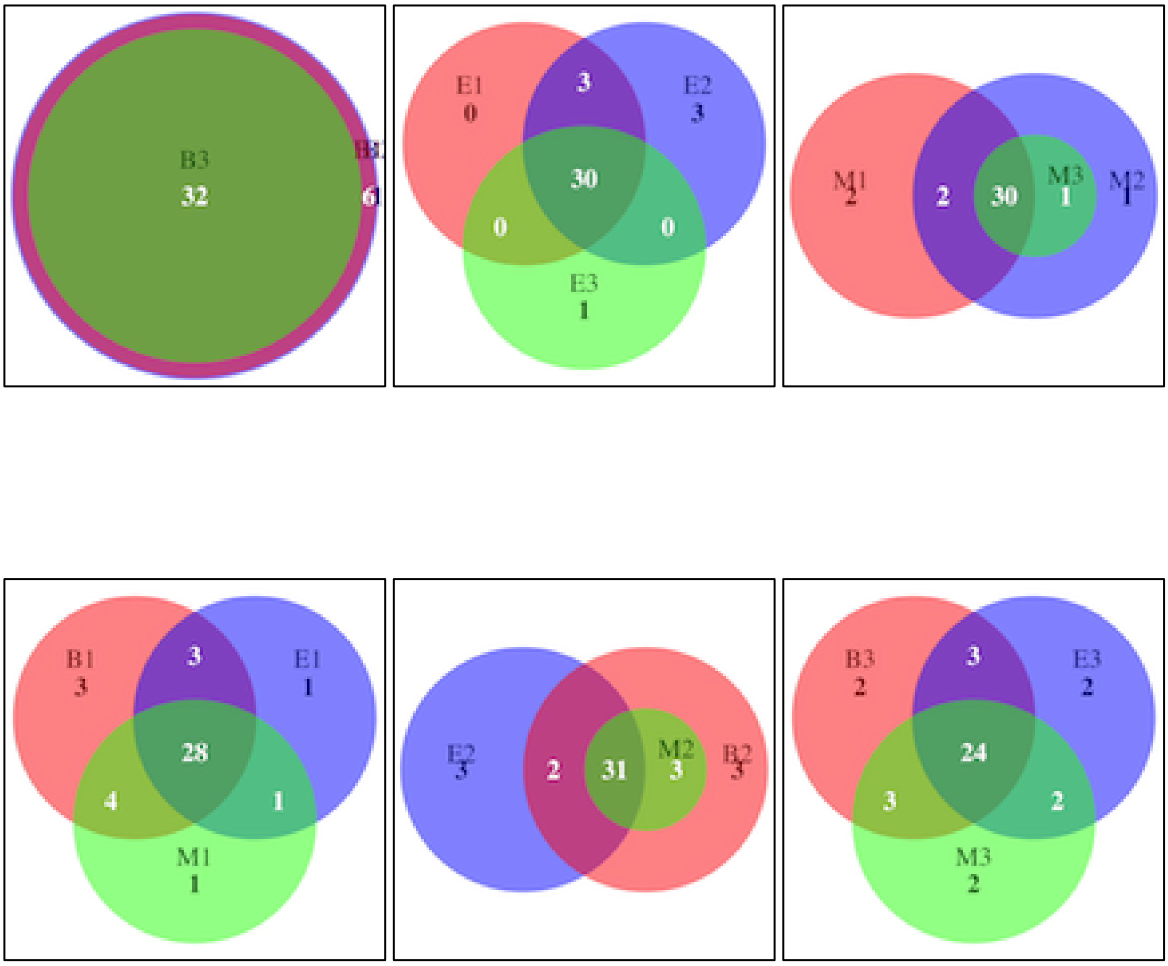

Supplementary Figure 1 Venn diagram showing number of shared and unique phyla among primers within the wetland (above) and among wetlands of the primers (below). The abbreviation used are as: B=Bhomra; E=EKW; M= Malencho; 1= 341b4_F/806_R; 2=N341b4_F/806_R; 3=Pro341F/Pro805R.

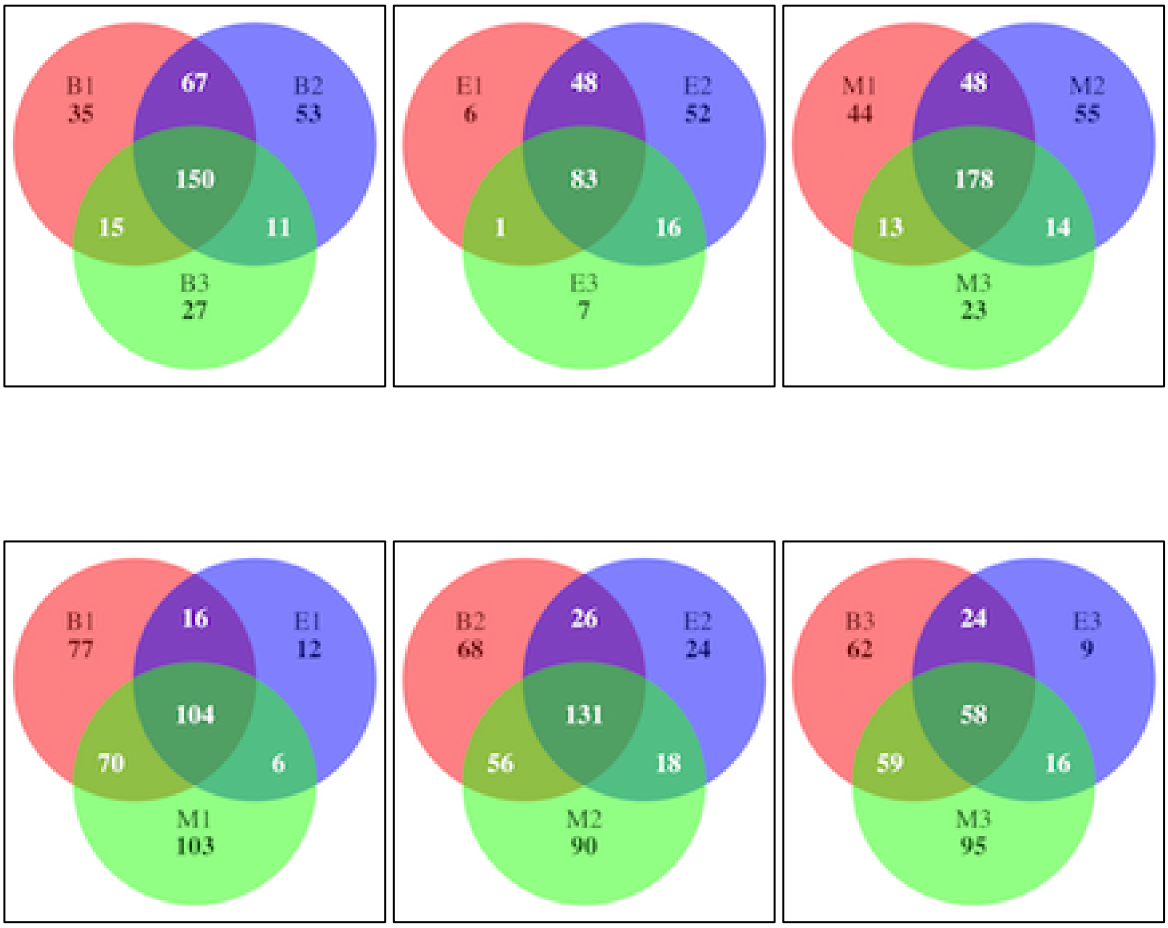

Supplementary Figure 2 Venn diagram showing number of shared and unique genera among primers within the wetland (above) and among wetlands of the primers (below). The abbreviation used are as: B=Bhomra; E=EKW; M= Malencho; 1= 341b4_F/806_R; 2=N341b4_F/806_R; 3=Pro341F/Pro805R.
